# Scaling Organic Electrosynthesis: The Crucial Interplay between Mechanism and Mass Transport

**DOI:** 10.1021/acscentsci.4c01733

**Published:** 2025-02-11

**Authors:** Zachary J. Oliver, Dylan J. Abrams, Luana Cardinale, Chih-Jung Chen, Gregory L. Beutner, Seb Caille, Benjamin Cohen, Lin Deng, Moiz Diwan, Michael O. Frederick, Kaid Harper, Joel M. Hawkins, Dan Lehnherr, Christine Lucky, Alex Meyer, Seonmyeong Noh, Diego Nunez, Kyle Quasdorf, Jaykumar Teli, Shannon S. Stahl, Marcel Schreier

**Affiliations:** † Department of Chemical and Biological Engineering, University of Wisconsin-Madison, Madison, Wisconsin 53706, United States; ‡ Department of Chemistry, University of Wisconsin-Madison, Madison, Wisconsin 53706, United States; § Chemical Process Development, Bristol Myers Squibb, 1 Squibb Drive, New Brunswick, New Jersey 08903, United States; ∥ Drug Substance Technologies, Process Development, Amgen, Inc., 1 Amgen Center Drive, Thousand Oaks, California 91320, United States; ⊥ Small Molecule Process Chemistry, Genentech, Inc., 1 DNA Way, South San Francisco, California 94080, United States; # Process Research & Development, AbbVie, 1401 Sheridan Road, North Chicago, Illinois 60064, United States; g Synthetic Molecule Design and Development, Eli Lilly and Company, Indianapolis, Indiana 46285, United States; h Process Chemistry, Chemical R&D, Pfizer Worldwide R&D, Eastern Point Road, Groton, Connecticut 06340, United States; i Process Research & Development, Merck & Co., Inc., Rahway, New Jersey 07065, United States; j Delivery Devices & Connected Solutions, Eli Lilly and Company, Lilly Capability Center India, Bangalore, Karnataka 560103, India

## Abstract

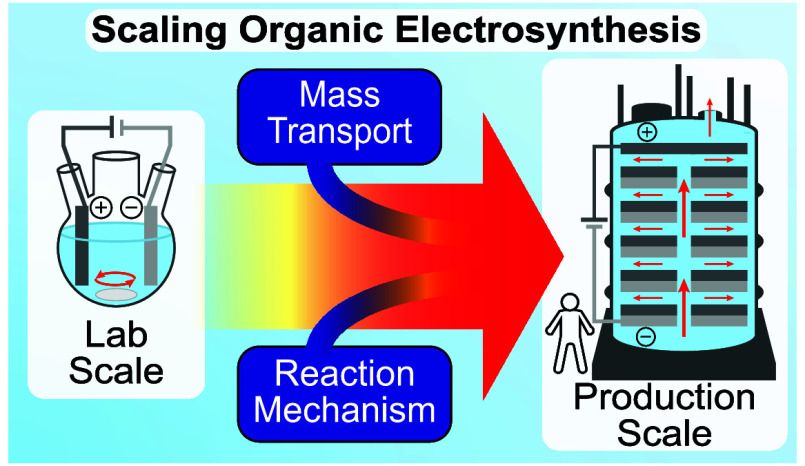

Organic electrosynthesis opens new avenues of reactivity and promises more sustainable practices in the preparation of fine chemicals and pharmaceuticals. The full value of this approach will be realized by taking these processes to the production scale; however, achieving this goal will require a better understanding of the influence of mass transport on reaction behavior and the interactions between reactive species and electrodes inherent to organic electrosynthesis. The limited options for cell geometries used on small scale limit elucidation of these features. Here, we show how advanced cell geometries allow us to control the interplay between reaction mechanism and mass transport, leading to improved performance of three modern organic electrosynthetic reactions. Each reaction shows a unique relationship with mass transport, highlighting the importance of understanding this relationship further to maximize the utility of organic electrosynthesis at scale.

## Abstract 2

In this work, we systematically investigate the relationship between mass transport and reaction mechanism, enabling the scale-up of organic electrosynthesis.

## Introduction

**Figure 1:**
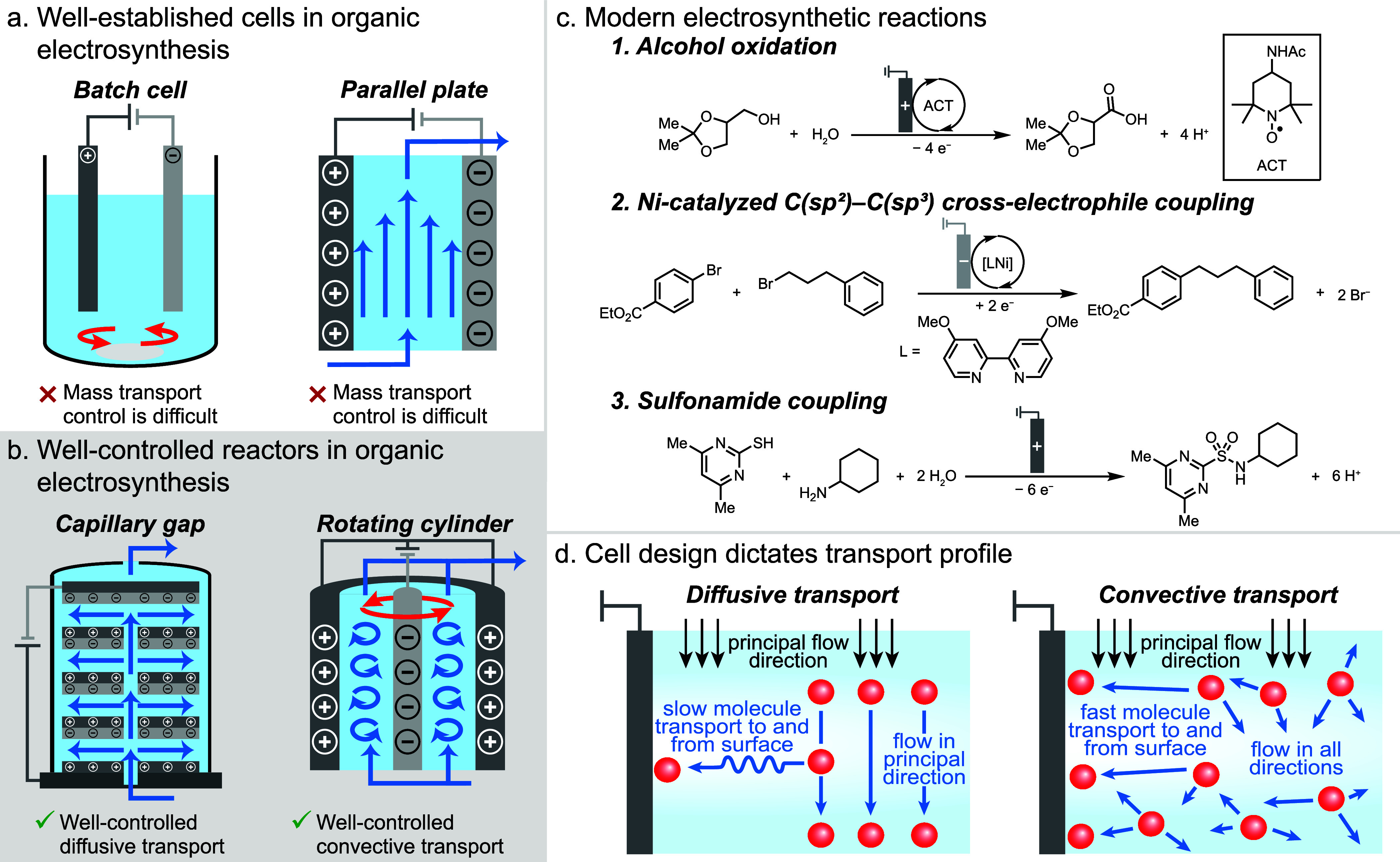
(a) Electrochemical cell configurations commonly used for electrosynthesis. (b) Engineered electrochemical reactors that support more controlled mass transport behavior. (c) Three prototype organic electrosynthesis reactions evaluated in this study. (d) Schematic illustration of the diffusive and convective transport regimes accessible with the capillary gap and rotating cylinder reactors.

Organic electrosynthesis represents a promising approach to access novel reactivity and improve sustainability in pharmaceutical and fine chemical synthesis.^1−7^ Realizing the potential of these methods will require robust strategies to operate these electrochemical processes at high rates at scales relevant for chemical production. Certain organic electrosynthesis processes have been scaled-up successfully, for example in parallel plate reactors or in capillary gap reactors.^2,5,8,9^ However, at these scales, phenomena beyond the mere reaction conditions become relevant in defining reaction outcomes.^10^ For example, it is well-known in the engineering field that the rate and selectivity of chemical reactions are heavily influenced by the rate of mass transport, which refers to how quickly molecules move through and within the reactor.^11^ This principle applies strongly to organic electrosynthesis, where the transport of substrates, products, and reaction intermediates to and from the electrode surface plays a crucial role in determining the reaction outcome. This feature is seldom considered in laboratory-scale studies, where general-use reactors, such as stirred batch cells or parallel plate flow cells, are commonly used (Figure 1a).^5,12−14^ These cells enable fast-paced research and are readily adapted to different reaction types, but they lack precise control over mass transport.^15^ Cells that can enable better mixing have been investigated, for example, through the use of static mixers or ultrasound,^15−18^ but the influence of mass transport on the outcome of organic electrosynthesis remains poorly understood. In this report, we probe the relationship between reaction pathways and mass transport using advanced cell designs (Figure 1b) and show how insights from these studies may be used to control the rate and selectivity of organic electrosynthesis reactions. Our findings demonstrate the crucial role of mass transport in organic electrosynthesis, and they provide insight into the types of reactors most suitable for scaling organic electrosynthesis processes.

The results outlined herein highlight the interplay between mass transport, which changes with different cell configurations, and the reaction mechanism, which varies with reaction type. Insights into the influence of mass transport on each of the tested reactions subsequently enabled the development of high-yield, single-pass, continuous-flow conditions for each reaction. Collectively, these results provide a crucial foundation for the future development of large-scale applications of organic electrosynthesis, illuminating the importance of understanding the role of mass transport in electrosynthesis.

## Results and Discussion

### Overview of Approach

We demonstrate and explain how mass transport influences the selectivity, rate, and even the feasibility of organic electrosynthetic reactions using three representative reaction classes of pharmaceutical interest (Figure 1c): (1) nitroxyl-mediated oxidation of alcohols to carboxylic acids,^19,20^ (2) reductive C(sp^2^)–C(sp^3^) cross-electrophile coupling (XEC) catalyzed by nickel,^21−24^ and (3) oxidative coupling of thiols and alkylamines to sulfonamides.^25^ These three reactions differ principally in their underlying mechanism and include both oxidative and reductive processes as well as both direct and indirect (i.e., mediated) electrolysis methods. In alcohol oxidation (Reaction 1), 4-acetamido-2,2,6,6-tetramethylpiperidine *N*-oxyl (ACT) mediates base-assisted oxidation of the alcohol to the aldehyde and then to the carboxylic acid. In Ni-catalyzed XEC (Reaction 2), nickel serves as a molecular electrocatalyst that undergoes electrochemical reduction steps at the cathode and chemical reactions with the two coupling partners. In sulfonamide coupling (Reaction 3), substrate oxidation involves a sequence of electrochemical and chemical reactions that generates the sulfonamide product. To probe the effects of mass transport on these reactions, we chose to employ cell geometries which allow us to exclusively foster either diffusive transport, where movement to and from the electrode surface is controlled by relatively slow Brownian motion, or convective transport, where bulk fluid motion results in fast movement of chemical components to and from the surface (Figure 1d).^26^ This approach allowed us to precisely control the rate at which chemicals interact with the electrode surface.

### Preliminary Assessment of Benchmark Reactions

We initiated our study by evaluating each of the three model reactions according to conditions previously reported in the literature in general-use laboratory-scale reactors.^19,25,27,28^ We conducted two of the reactions, alcohol oxidation (Reaction 1) and Ni-catalyzed XEC (Reaction 2), in stirred batch cells, and we conducted sulfonamide coupling (Reaction 3) by recirculating the reaction solution through a parallel plate reactor.

**Figure 2:**
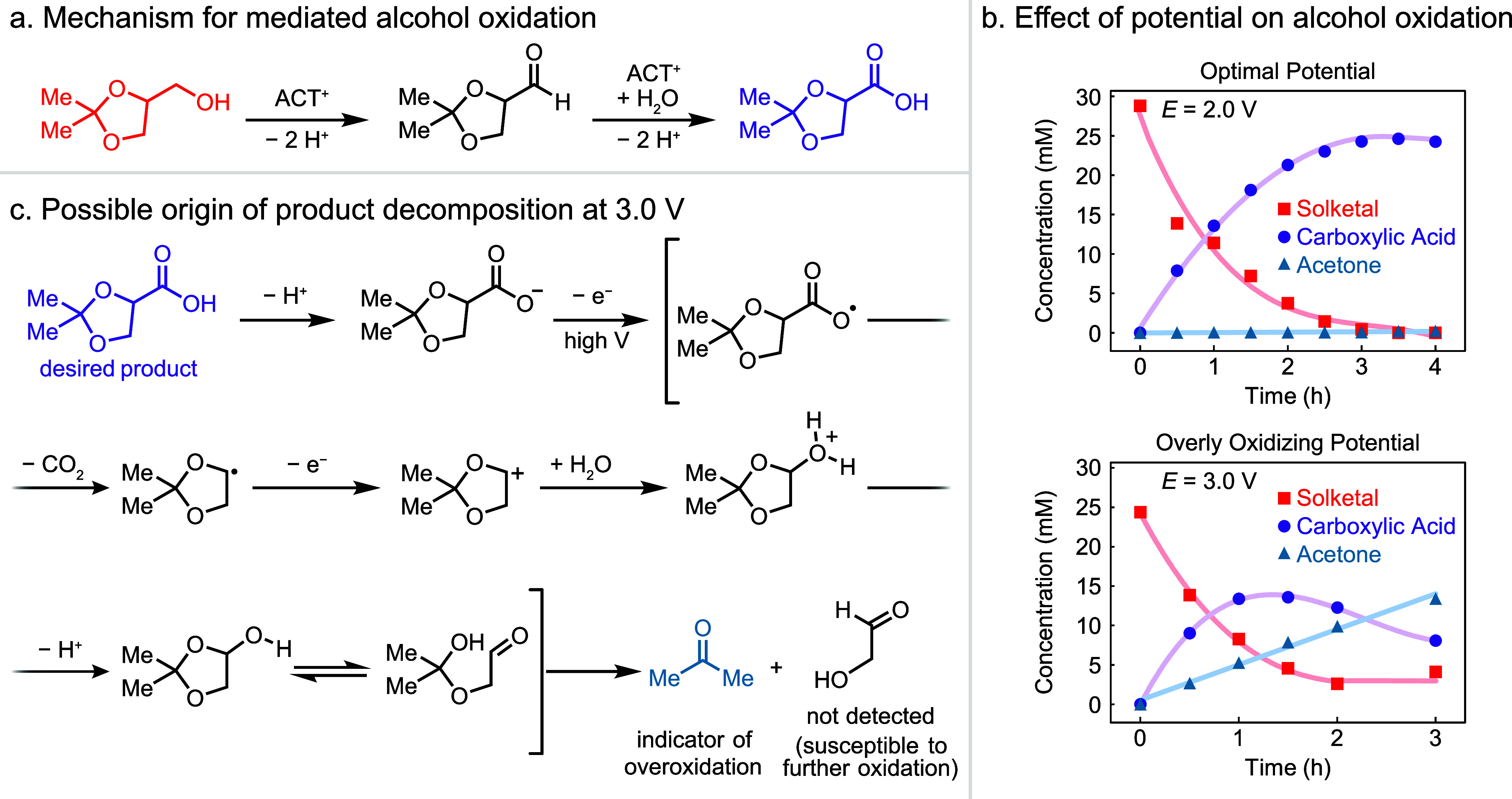
(a) Two oxidative steps in the mediated oxidation of alcohols. (b) Time-courses of solketal oxidation in a batch cell at *E*_cell_ = 2.0 V and *E*_cell_ = 3.0 V. Reaction conditions: 25 mM solketal, 5 mol % ACT, 0.1 M NaHCO_3_, 0.1 M Na_2_CO_3_ in H_2_O. Lines serve as a guide to the eye. (c) Mechanistic steps leading to product decomposition to acetone at higher driving forces.

The alcohol oxidation (Reaction 1) uses an electroactive nitroxyl mediator, ACT, to catalyze oxidation of a primary alcohol, solketal, to a carboxylic acid via sequential oxidation of the alcohol and the intermediate aldehyde hydrate (Figure 2a).^19^ This reaction is well-studied and exhibits fast kinetics, with ACT exhibiting high turnover frequencies.^20,29−31^ In a beaker-type cell containing a concentric array of alternating polarity electrodes (seven graphite rod anodes and seven stainless steel rod cathodes)^32^ at a constant applied cell potential of 2.0 V, solketal cleanly converted to the corresponding carboxylic acid in 85% yield (Figure 2b). At a higher applied cell potential of 3.0 V, we observed a faster rate of substrate consumption; however, these conditions led to a reduced yield, reaching a maximum of 56% at 1 h before decreasing at a longer reaction time. This result arose from product decomposition and formation of acetone, attributed to oxidative decarboxylation of the product (Figure 2c).^33,34^ These findings indicate that the applied potential must be constrained to avoid overoxidation.

**Figure 3:**
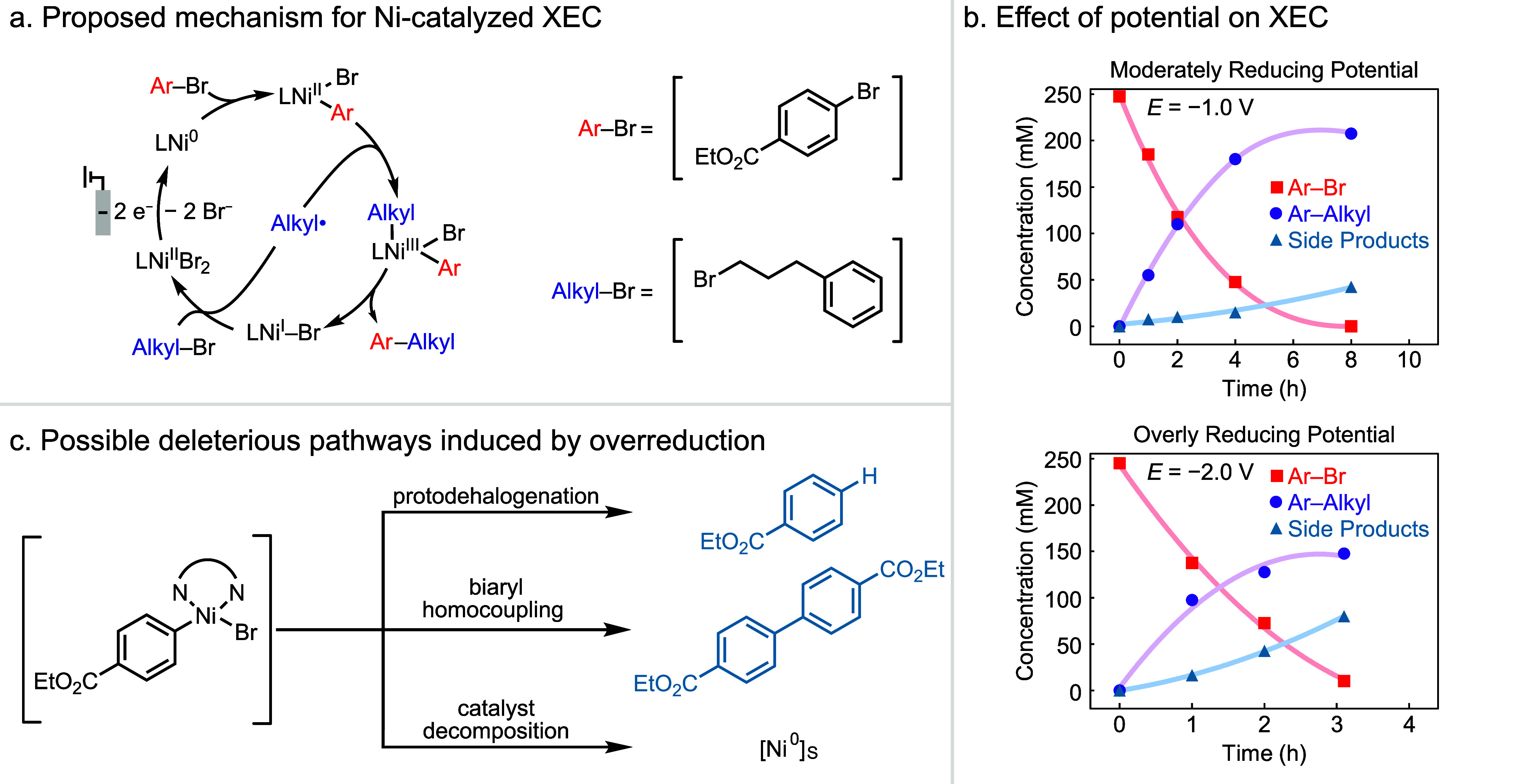
(a) Proposed catalytic mechanism for Ni-catalyzed XEC of aryl and alkyl halides. (b) Yield of product and byproducts at two different driving forces in the Ni-catalyzed XEC reaction. Reaction conditions: 0.25 M Ar–Br, 0.33 M Ar–Alkyl, 5 mol % NiBr_2_·3H_2_O, 5 mol % 4-4'-dimethoxy-2-2'-bipyridine (dMeObpy), and 0.2 M NaI in dimethylacetamide (DMA). Lines serve as a guide to the eye. (c) Deleterious outcomes of over-reduction of Ni intermediates.

In the reductive Ni-catalyzed XEC (Reaction 2), electrochemical reduction of the Ni^II^ precatalyst to Ni^0^ initiates a series of chemical and electrochemical steps that afford the C–C-coupled product (Figure 3a). The Ni catalyst is similar to ACT in that it mediates the reaction, but unlike alcohol oxidation, this reaction undergoes a series of slower off-electrode steps. Previous work suggests that key chemical steps include oxidative addition of the Ar–Br, radical generation from the Alkyl–Br, alkyl radical addition to the arylnickel intermediate, and C–C reductive elimination (Ar–Alkyl).^28,35−38^ Electrons from the cathode initiate the catalytic reaction and cycle the Ni catalyst. We performed reactions in stirred vials equipped with a graphite rod cathode and zinc plate anode under a constant cell potential of −1.0 V (measured cathode to anode), and we obtained the desired product in 88% yield (Figure 3b). At a cell potential of –2.0 V, the reaction selectivity dropped, and we observed increased formation of side products, arising from proto-dehalogenation and electrophile homocoupling. These results match previous studies that have shown that overreduction of Ni complexes at more negative potentials or excessive current density can lead to these side products (Figure 3c).^22,28,39^ Thus, a high product yield in the Ni-catalyzed XEC reaction relies on controlling the applied potential, similar to observations made in the alcohol oxidation reaction.

**Figure 4:**
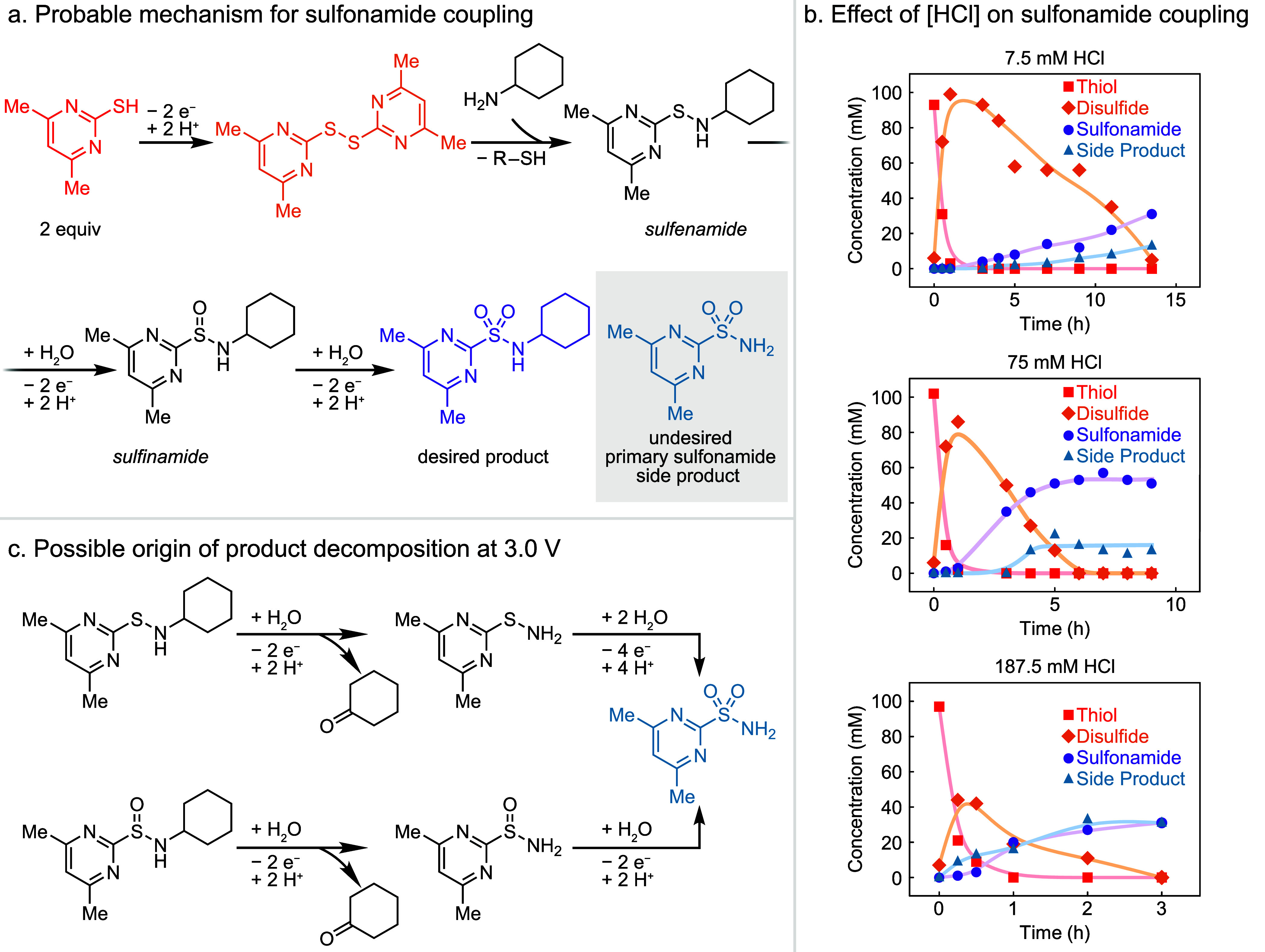
(a) Oxidative processes leading to product and byproduct in sulfonamide coupling with putative intermediates. (b) Three time courses in a recirculated parallel plate cell at three different concentrations of HCl. Reaction conditions: 0.1 M thiol, 0.15 M cyclohexylamine, 0.01 M tetrabutylammonium tetrafluoroborate (TBA BF_4_) in 12.3:1 v/v acetonitrile:water (MeCN:H_2_O) with 0.1, 1, and 2.5 M HCl. Lines serve as a guide to the eye. (c) Two plausible mechanistic origins of the strong dependence of yield on acid. Additional possible mechanisms are given in the SI.

The original sulfonamide coupling reaction (Reaction 3) was conducted in an electrochemical flow reactor^40^ similar to a parallel plate reactor. Unlike alcohol oxidation and Ni-catalyzed XEC, sulfonamide coupling involves direct electron transfer without a mediator. The reaction is proposed to be initiated by direct electrochemical oxidation of the thiol to a disulfide, and then features a sequence of oxidative coupling steps involving the disulfide, amine, and water, proceeding through sulfenamide and sulfinamide intermediates (Figure 4a).^25,41,42^ The primary sulfonamide, lacking the *N*-alkyl group, is a major side product of the reaction. At 3.4 V in a parallel plate cell equipped with a graphite anode and stainless steel cathode, we formed the desired product in 51% yield, with a 13% yield of the primary sulfonamide. The acid concentration played an important role in the reaction, with increasing and decreasing concentrations leading to lower product yield and elevated yields of the side product (Figure 4b). This outcome mirrors previous observations, with low yields at higher and lower loading of acid.^25^ We postulate that local concentrations of acid formed at the anode protect the amine substrate and/or intermediates from dealkylation (Figure 4c).^43−45^ The data further suggest that maintaining control over the acid concentration is essential to achieving high product yields and minimizing side product formation during sulfonamide coupling. Reaction 1 also generates acid during alcohol oxidation, but this reaction is much less sensitive to acid.^29^

The different mechanisms associated with each of these reactions raise the possibility that they will exhibit a different dependence on the transport behavior of reagents and catalysts. To explore such behavior, we could not rely on the typical laboratory cell designs as they do not provide rigorous, uniform control over the mass transport to and from the electrode. Instead, we turned to two different reactor designs that enable a high degree of control.

### Overview of Reactor Designs and Flow Properties

**Figure 5:**
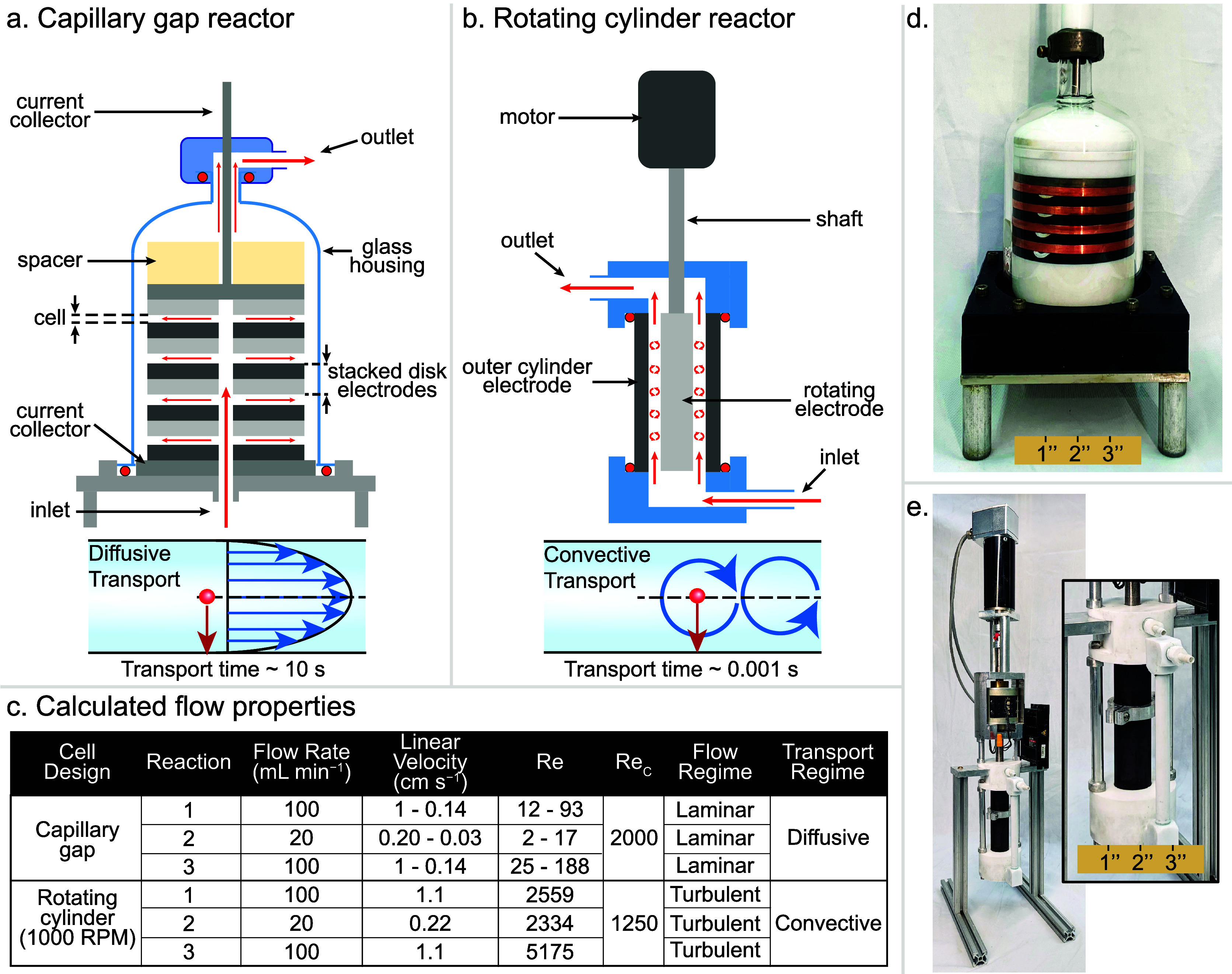
Schematics of (a) capillary gap and (b) rotating cylinder with the associated transport regime. (c) Flow properties, calculated Reynolds numbers, and critical Reynolds numbers for each experiment. Image of the (d) capillary gap reactor and (e) rotating cylinder reactor.

The two flow reactors employed here include a capillary gap (CG) reactor and a rotating concentric cylinder (RC) reactor. These reactors foster either diffusive or convective transport and thus provide a foundation for investigating the influence of mass transport on the electrosynthetic reactions introduced above. Our reactors were adapted from established designs, and may be summarized as follows (full details on reactor assembly are provided in the Supporting Information). In the CG reactor (Figure 5a),^12,46−50^ the solution is delivered upward through the center of electrode discs and flows radially outward between stacked plates. The electrodes within the stack are separated by a small gap (≤1 mm), and the solution exhibits laminar flow, resulting in exclusively diffusive transport between the bulk solution and electrode surface. The RC reactor consists of a solid cylindrical electrode that rotates inside a concentric tubular electrode that remains static.^51−55^ The solution flows upward through the cell (Figure 5b), and rotation of the internal cylinder entrains the fluid, creating turbulence that promotes convective transport between the bulk and the electrode surfaces.

To assess the flow profile in each reactor, Reynolds numbers (Re) were determined under the employed operating conditions (Figure 5c). The Re number is used to assess the flow regime at a set of given reactor and flow conditions through comparison to the critical Reynolds number (Re_c_), which is determined by the reactor geometry. Typically, an Re value less than the Re_c_ indicates laminar flow, while Re values greater than Re_c_ indicate turbulence.^26^ The calculated Re and Re_c_ values for the two reactors support the assignment of laminar flow and diffusive transport in the CG reactor and turbulent flow and convective transport in the RC reactor. Linear velocities, which have been reported to impact reaction outcome,^56^ are given with the calculated Re and Re_c_ values in Figure 5c. Computational fluid dynamics analysis of each electrochemical cell further supports this analysis (Figure S1). In the CG reactor, the velocity is highest at the midpoint between the parallel electrodes, as expected for laminar flow.^26^ In contrast, flow within the RC reactor features the formation of vortices that leads to rapid transport of the bulk solution between the two electrodes,^51,57^ consistent with the assignment of turbulent mixing derived from the Reynolds numbers.

### Comparison of Different Reactions in the Capillary Gap and Rotating Cylinder Reactors

**Figure 6:**
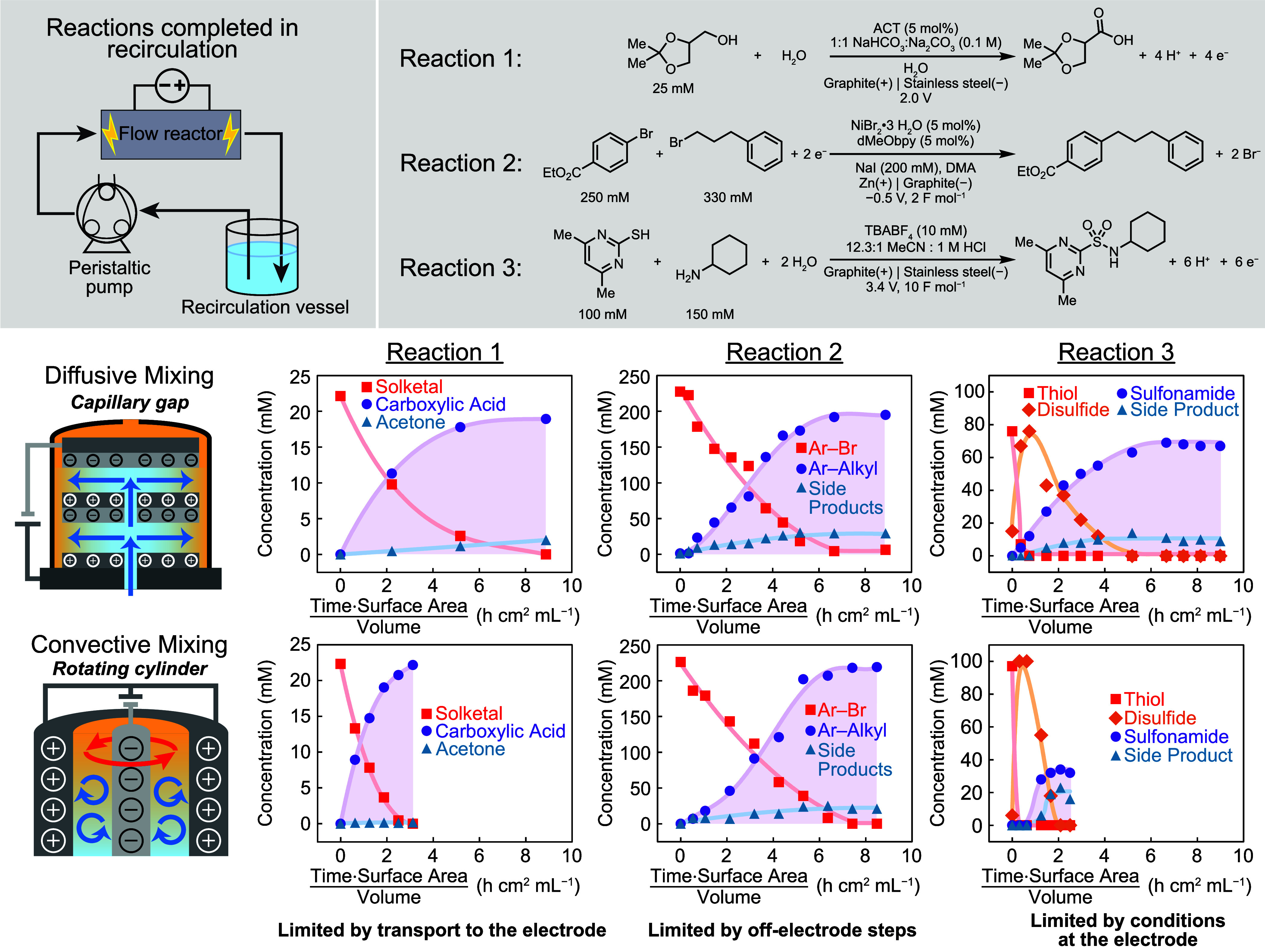
Time courses for each of the three reactions in the capillary gap and rotating cylinder reactors. We report substrate concentration *vs* time·surface area/solution volume to normalize for variations in surface area. Lines serve as a guide to the eye.

We examined each of the three reactions in both the CG and the RC reactors in a recirculating-flow configuration (Figure 6). We report substrate concentration *vs* time·surface area/solution volume to normalize for variations in surface area. Additional experimental details, including information on the electrode materials that can be used in these reactors, are provided in the SI.

We found that the alcohol oxidation reaction (Reaction 1), conducted at a constant cell potential of 2.0 V, exhibited a much better performance in the RC reactor relative to the CG reactor. The reaction reached completion much more rapidly in the RC reactor, reaching 100% substrate conversion nearly three times faster than in the CG reactor. We also observed improved yield in the RC reactor at 89%, compared to 76% in the CG. We attribute the increase in rate to the synergy between rapid mass transport in the RC reactor and fast solution kinetics of the ACT-mediated alcohol oxidation.^19^ Under convective transport, rapid regeneration of the reduced mediator at the electrode surface and subsequent rapid chemical transport of oxidizing equivalents away from the surface to substrates in the bulk result in a high production rate.

The high mass transport fostered by the RC reactor enables a faster reaction rate while maintaining mild potentials, ensuring that the product does not undergo undesired overoxidation to acetone. In contrast, under diffusive transport in the CG reactor, the ACT mediator takes longer to move between the electrode surface and the bulk solution, substantially decreasing the production rate. The advantages afforded by the fast rates with which ACT oxidizes the alcohol substrate are therefore limited by the slow mass transport within the CG reactor.

We found that the Ni-catalyzed XEC (Reaction 2) exhibited minimal difference in rate between the two reactors but reached a higher yield in the RC reactor at 97% as compared with 86% in the CG reactor. We performed the reaction at a constant cell potential of −0.5 V (measured cathode to anode) to minimize side product formation, which formed in significant quantity at the more negative applied potential of −1.0 V (Figure S7). The reaction reached completion at similar rates in the CG and RC reactors, especially when compared to the 3-fold reduction in rate recorded for solketal oxidation in the RC. We attribute the modest improvement in the XEC yield in the RC reactor to better transport of the Ni catalyst to and from the electrode surface, promoting slightly faster catalytic cycling of Ni and limiting off-cycle side product formation. However, relatively slow off-electrode chemical steps mediated by the Ni catalyst limit the benefits that can be accessed from convective transport. These observations show that for mediated electrochemical reactions, the catalyst must promote sufficiently fast chemical steps to benefit from improved mass transport. For alcohol oxidation, we report synergies between fast mass transport and fast ACT kinetics, while here the slower chemical steps that govern this reaction class appear to show a weaker dependence on mass transport.

The sulfonamide coupling reaction (Reaction 3) exhibited a dependence on mass transport different from that of the other two reactions. Under laminar flow in the CG reactor, we observed product formation at 74% yield with modest formation of the undesired side product (10%). Under convective mixing in the RC reactor, the reaction was noticeably more rapid, but the yield was much lower at 33% and side product formation was more significant, reaching 22%. In short, sulfonamide coupling performs worse under increased mass transport. This unusual outcome resembles behavior observed with certain dimerization reactions evaluated with rotating ring disk electrodes.^58^

We attribute these observations to the role of proton sources in the reaction, related to our preliminary findings in the parallel plate reactor discussed above (Figure 4b). We hypothesize that acid generated through the direct oxidations of substrates and intermediates at the anode can protect intermediates from undesired oxidative decomposition (i.e., via dealkylation of the amine fragment). Although protons will eventually be consumed through H_2_ evolution at the cathode, the relatively slow diffusion of protons away from the anode surface in the CG reactor allows for the maintenance of a locally acidic environment at the anode, resulting in improved reaction performance. In contrast, efficient convection in the RC reactor homogenizes the environment at both electrode surfaces with the bulk due to fast transport of generated species away from the electrode surfaces. The improved mass transport results in approximately 3-fold faster consumption of the reagents, but the resulting lower proton activity at the anode leads to increased formation of side product and lower product yield. In addition, the reaction proceeds best in both reactors with excess charge passed (10 F mol^–1^), potentially indicating that charge is being cycled unproductively between the cathode and anode in the rotating cylinder reactor. These observations suggest that the reaction microenvironment created under diffusive transport is more advantageous to this reaction than the rate benefits associated with convective transport. The microenvironment at the anode is of particular importance during sulfonamide coupling since numerous direct electrochemical oxidation steps are required to form the sulfonamide product. This reactivity contrasts observations with the mediated reactions, which promote chemical transformations in bulk solution and show higher rates with increased mass transport.

Collectively, these observations highlight the important interplay between the reaction mechanism and mass transport. For alcohol oxidation, fast chemical steps allow the reaction to benefit significantly from the faster mass transport available in the RC reactor. The nickel-catalyzed reaction steps are too slow to show the same improvement in reaction rate from faster mass transport, resulting in nearly the same performance in both reactors. The sulfonamide coupling reaction, on the other hand, benefits from slow proton diffusion from the anode to cathode exhibited by the CG reactor as the proton gradient enhances reaction selectivity by ensuring a microenvironment that promotes product rather than side-product formation. The sulfonamide coupling data are particularly noteworthy, because they show that not all electrosynthetic reactions will benefit from increased mass transport.

### Development of Single-Pass Flow Reaction Conditions

**Figure 7:**
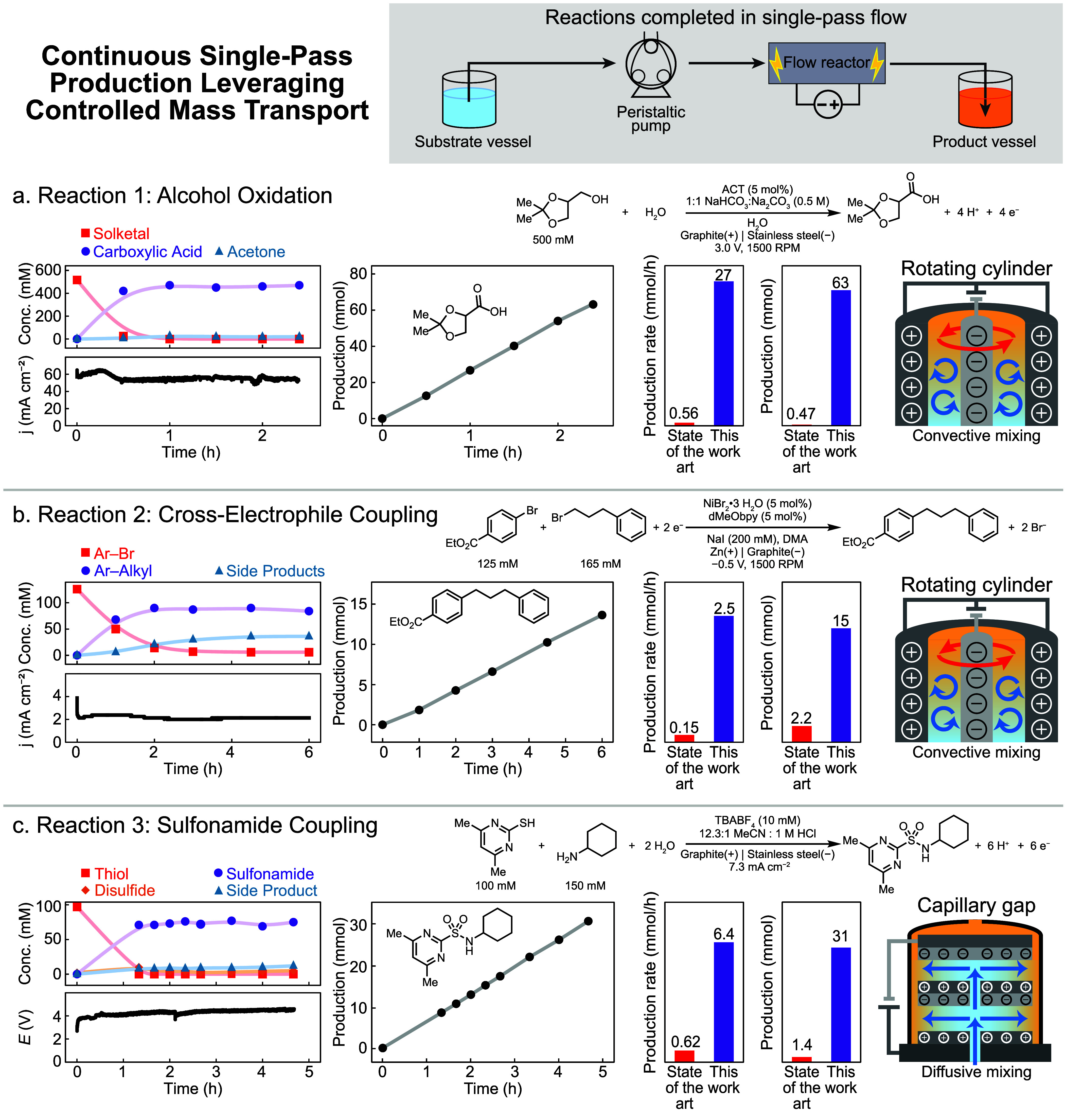
Single-pass production in the associated highest performing transport regime for (a) alcohol oxidation, (b) cross-electrophile coupling, and (c) sulfonamide coupling. Production rate values correspond to absolute production rates to reflect the scale at which the reactions were carried out. Details on production rate calculations can be found in the SI. Lines serve as a guide to the eye.

Having defined the optimal transport conditions and optimal reactor designs for the three reactions, we sought to use this insight to enable rapid single-pass formation of each product. In single-pass flow chemistry, reactors are operated under conditions that allow for high substrate conversion to the desired product in a single pass through the reactor, allowing for continuous production of the compound of interest. We performed each reaction in its associated optimal reactor: solketal oxidation and XEC in the RC reactor and sulfonamide coupling in the CG reactor. For solketal oxidation, the higher mass transport in the RC reactor allowed the reaction to be performed at a higher driving force, 3.0 V, while still limiting the product overoxidation into acetone. Previous work on optimizing alcohol oxidation reactions has prioritized recirculated flow processes that increase the mass transport by increasing the flow rate,^20^ but this approach is incompatible with high single-pass yields. Here, the RC reactor can decouple mass transport from the flow rate, allowing high production rates for alcohol oxidation to be achieved in a continuous single-pass flow configuration. We formed over 50 mmol product while maintaining 90% yield over 2 h (Figure 7a). Existing precedent for single-pass electrochemical alcohol oxidation provides significantly less material at a much lower rate due to reliance on diffusive mixing in a microfluidic electrolytic cell (similar to a standard parallel plate cell).^59^ When performing Ni-catalyzed XEC in the RC reactor under single pass flow, we formed more than 15 mmol of cross-coupled product in 70% yield over 6 h (Figure 7b). This represents a 16-fold increase in production rate as compared to a previous single-pass production study employing a parallel plate cell.^21^ Interestingly, the previous study yielded very similar yield to our work, albeit by using higher temperatures (75 °C), a 3-dimensional electrode, and twice the Ni catalyst loading. The similar selectivity observed in these two systems reinforces the notion that the outcome of the XEC reactions strongly depends on the intrinsic performance of the Ni catalyst. Finally, for sulfonamide coupling, we leveraged highly controlled diffusive transport conditions in the CG reactor to carry out the reaction at 74% yield at constant current, and we formed over 30 mmol of sulfonamide product in less than 5 h of electrolysis with minimal byproduct formation (Figure 7c). These results represent an order-of-magnitude increase in productivity over that accessed in the previous microflow parallel plate cell design.^25^ In summary, these results show that understanding and controlling mass transport provides the foundation for the development of high-yield, single-pass, continuous electrosynthetic processes that can access significant improvements in throughput and productivity.

## Conclusion

When faced with poor performance or slow rates in organic electrosynthesis reactions, common adjustments include changes to reactant concentrations, increasing the electrode surface area, decreasing interelectrode gaps, or changing the applied potential. Many of these adjustments alter how and when molecules in solution interact with one or both of the active electrode surfaces. Thus far, systematic insights into these mass transport effects have been lacking. In the present study, the use of electrolysis cells engineered to control mass transport behavior has enabled us to probe the relationship between mass transport and reaction outcomes. Specifically, we used capillary gap and rotating concentric cylinder reactors to induce diffusive and turbulent reaction conditions, respectively, and we used these well-defined flow environments to gain insight into the impact of mass transport on three representative organic electrosynthesis reactions. Our findings showed that different organic electrochemical reaction mechanisms benefit from different mass transport environments. For some reactions (e.g., mediated alcohol oxidation), high mass transport leads to improved reaction rates and selectivity. High rates of mass transport are not universally beneficial, however. Some reactions (e.g., oxidative sulfonamide coupling) significantly improved under low mass transport conditions. In the case studied here, this behavior arises from the need to accumulate protons near the electrode, creating a beneficial acidic microenvironment at the electrode surface. A third class of reactions (e.g., Ni-catalyzed cross-electrophile coupling) is only minimally impacted by fluid flow patterns and mass transport and is instead controlled by the slow intrinsic kinetics of the molecular catalyst. We leveraged this insight to demonstrate the rational design of single-pass synthesis approaches for all three reaction types, enabling the development of effective single-pass continuous processes for all three reactions. These results show how insights into the role of mass transport are fundamentally important to organic electrosynthetic reactions and processes. Rational control of mass transport is thus an important and underappreciated “knob” that can be turned to alter or improve the performance of electrosynthesis reactions as a function of the underlying mechanism. This understanding is important for lab-scale research but will be especially crucial in future applications of large scale electrosynthetic processes.

## Supporting Information Available

Information on the materials and methods, including details of the capillary gap and rotating cylinder reactors and reactor cleaning procedures; electrode characterization; flow regimes and calculation of Reynolds numbers; mass transport calculations and computational fluid dynamics simulations of the capillary gap and rotating cylinder reactors; experimental procedures; and calculations of production rates relating to Figure 7 (PDF)
