# Supplementary material for: Scaling Organic Electrosynthesis: The Crucial Interplay between Mechanism and Mass Transport
Source: ACS Cent Sci. 2025 Feb 11;11(4):528–38. doi: 10.1021/acscentsci.4c01733 (PMC12022915; doi:10.1021/acscentsci.4c01733)
Supplement: Supplementary file 1 — oc4c01733_si_001.pdf [file oc4c01733_si_001.pdf]

Supporting Information:

Scaling Organic Electrosynthesis: The Crucial Interplay between Mechanism and Mass Transport

Zachary J. Oliver[a], Dylan J. Abrams[a,b], Luana Cardinale[b], Chih-Jung Chen[a], Gregory L. Beutner[c], Seb Caille[d], Benjamin Cohen[c], Lin Deng[e], Moiz Diwan[f], Michael O. Frederick[g], Kaid Harper[f], Joel M. Hawkins[h], Dan Lehnherr[i], Christine Lucky[a], Alex Meyer[a], Seonmyeong Noh[a], Diego Nunez[a], Kyle Quasdorf[d], Jaykumar Teli[j], Shannon S. Stahl[b], Marcel Schreier[a,b]**

[a] Department of Chemical and Biological Engineering, University of Wisconsin-Madison, Madison, Wisconsin 53706, United States

[b] Department of Chemistry, University of Wisconsin-Madison, Madison, Wisconsin 53706, United States

[c] Chemical Process Development, Bristol Myers Squibb, 1 Squibb Drive, New Brunswick, New Jersey 08903, United States

[d] Drug Substance Technologies, Process Development, Amgen, Inc., 1 Amgen Center Drive, Thousand Oaks, California 91320, United States

[e] Small Molecule Process Chemistry, Genentech, Inc, 1 DNA Way, South San Francisco, California 94080, United States

[f] Process Research & Development, AbbVie, 1401 Sheridan Road, North Chicago, Illinois 60064, United States

[g] Synthetic Molecule Design and Development, Eli Lilly and Company, Indianapolis, Indiana 46285, United States

[h] Process Chemistry, Chemical R&D, Pfizer Worldwide R&D, Eastern Point Road, Groton, Connecticut 06340, United States

[i] Process Research & Development, Merck & Co., Inc., Rahway, New Jersey 07065, United States

[j] Delivery Devices & Connected Solutions, Eli Lilly and Company, Lilly Capability Center India, Bangalore 560103, Karnataka, India

Table of Contents

Materials and Methods 4

General Considerations 4

Capillary Gap Cell 4

Rotating Cylinder Cell 5

Electrode Materials 5

Electrode and Reactor Parts Cleaning Procedure 5

Flow Regimes and the Reynolds Number 7

Mass Transport Calculations and Computational Simulations 9

Particle Mean Transport Time Calculations 9

Computational Simulations 9

Solketal Oxidation 10

Batch Potential Variation Experiments (Figure 2) 10

General Procedure – Capillary Gap Cell (Figure 6) 10

General Procedure – Rotating Cylinder Cell (Figure 6) 11

Current Densities for Runs in Recirculation 11

Runs in Recirculation at Varying Flowrates 11

General Procedure – Rotating Cylinder Cell at Single-Pass (Figure 7) 12

Cross-Electrophile Coupling 13

Batch Potential Variation Experiments (Figure 3) 13

General Procedure – Capillary Gap Cell (Figure 6) 13

General Procedure – Rotating Cylinder Cell (Figure 6) 14

Current Densities for Runs in Recirculation 14

Data for XEC Performed in Recirculation at -1.0 V 15

General Procedure – Rotating Cylinder Cell at Single-Pass (Figure 7) 15

Sulfonamide Coupling 17

Parallel Plate Acid Concentration Variation Experiments (Figure 4) 17

General Procedure – Capillary Gap Cell (Figure 6) 17

General Procedure – Rotating Cylinder Cell (Figure 6) 18
Current Densities for Runs in Recirculation.. 18
General Procedure – Capillary Gap Cell in Single-Pass (Figure 7)................................ 19
Primary Sulfonamide Side Product Formation.. 19
Water Oxidation for Sulfonamide Coupling.. 19

Electrode Characterization.. 21

Electrochemical Surface Area (ECSA)... 21
Charge Transfer Resistance 26

Calculating Production Rates.. 34
Literature Reports at Single-Pass.. 34
References.. 35
Representative NMR Spectra.. 37

Materials and Methods

General Considerations

The following chemicals were purchased from the indicated vendor and used as received: Solketal (97%, Thermo Scientific), 4-acetamido-TEMPO free radical (ACT) (≥98%, Thermo Scientific), $NaHCO_3$ (ACS grade ≥99.7%, VWR), anhydrous Na_2CO_3 (ACS grade 99.5%, VWR), 4,6-dimethyl-2-mercaptopyrimadine (98.0%, TCI), cyclohexylamine (≥98%, Thermo Scientific), tetrabutylammonium tetrafluoroborate (≥98.0%, TCI), hydrochloric acid (ACS reagent grade, 37%, Sigma Aldrich), ethyl-4-bromobenzoate (≥98%, Thermo Scientific), 3-phenylpropyl bromide (≥97.0%, TCI America), sodium iodide (ACS grade ≥99.5%, Thermo Scientific), nickel (II) bromide trihydrate (98%, Thermo Scientific), 4,4'-dimethoxy-2,2'-bipyridine (98%, Ambeed), 1,3,5-trimethoxybenzene (99%, Thermo Scientific), dimethyl sulfoxide (99.9%, Sigma Aldrich). Solvents acetonitrile (≥99.9%, Sigma Aldrich), N,N-dimethylacetamide (DMA) (99%, Thermo Scientific), and N,N-dimethylformamide (DMF) (anhydrous, 99.8%, Sigma Aldrich) were used as received and all water was MilliQ grade. Reagents and solvents used for nickel reactions were stored under an atmosphere of N_2.

All ^1H Nuclear magnetic resonance (NMR) spectroscopy was performed on a Bruker Avance-400 in D_2O (99.9 atom% D, Sigma Aldrich), CD_3CN (99.8 atom% D, Thermo Scientific Chemicals), and $CDCl_3$, (99.8 atom % D, Sigma Aldrich) which were all used as received. Flow was performed using a Masterflex peristaltic pump (Cole-Parmer, Masterflex, L/S, Digital Pump Drive, 07525-20) in Tygon Chemical L/S 14 Masterflex tubing. 1/4 NPT and 1/8 NPT PTFE fittings were purchased from Swagelok for fluid connections. Electrochemical reactions were performed using an Agilent 6032A power supply and data was recorded using National Instruments LabView 2021.

A beaker was used to collect solution that was pumped out of the cells and recirculate it back into the reactor. For all single-pass flow experiments, fresh solution was flowed directly into the flow cells and no solution was recirculated. Charge transfer resistance measurements were completed after each reaction under recirculation in the capillary gap and rotating cylinder reactor.

Capillary Gap Cell

The capillary gap cell was designed and manufactured in-house based on previous designs.[1,2] Graphite electrode plates were purchased from and machined by EDM Sales Inc (PGM-5). Stainless steel (corrosion-resistant 316 grade) and zinc (99%) plates were purchased from McMaster-Carr and machined in-house. Electrode plates were stacked on PTFE electrode posts (1/4-20 threaded, McMaster-Carr) that were threaded into the base plate in the following manner. An anode plate was first placed onto a titanium base plate, followed by a 1 mm PTFE spacer (McMaster-Carr, machined in-house) on each electrode post, and then followed by a cathode plate. This was repeated four times so that four individual electrode pairs were included in the complete cell. Electrical connection to the electrode plates was achieved with titanium plates (McMaster-Carr, machined in-house) placed at the bottom and top of the electrode stack. A titanium rod (McMaster-Carr, machined in-house) was threaded into the top titanium plate to establish electrical connection to the outside of the reactor. The reactor was housed in a glass dome, made in-house from glass reactor housing and process pipe (Ace Glass Incorporated). PTFE spacers (McMaster-Carr, machined in-house) were used to fill in empty space of the glass reactor housing. The reactor

was supported on a base (machined in-house) made from two PTFE parts (McMaster-Carr), an aluminum plate (McMaster-Carr), and aluminum feet (McMaster-Carr, machined in-house). The glass reactor housing was fixed to the base by connecting the two PTFE parts together and was kept leak-tight with a PTFE-coated rubber O-ring (McMaster-Carr). At the top of the reactor, process pipe coupling (Ace Glass Incorporated) connected the glass reactor housing to a PTFE cap (McMaster-Carr, machined in-house).

The electrodes in the capillary gap reactor were circular disks with of 3.812" diameter and were 0.25" thick. This resulted in 74 cm^2 inner electrode surface area. The interelectrode gap in this reactor was 1 mm.

Rotating Cylinder Cell

The rotating cylinder reactor was designed and constructed in-house. Graphite electrodes were purchased from and machined by EDM Sales Inc (PGM-5) with additional machining completed in-house. Stainless steel (316 grade) and zinc (99%) electrodes were purchased from McMaster-Carr and machined in-house. The electrodes were fixed between two PTFE electrode housing parts (McMaster-Carr, machined in-house) and kept leak-tight with PTFE O-rings (Grainger). The inner cylinder was connected to a 5/8" rotator shaft made from titanium (McMaster-Carr, machined in-house) by a threaded rod (McMaster-Carr). A brushless DC motor (167131, 400 W, Maxon Motors) and an ESCON 70/10 servo controller (422969) from Maxon Motors were used for controlling rotation and a shaft coupling (Grainger) connected the motor to the shaft. Electrical connection to the rotating shaft was established using a slip-ring (Rotary Systems, 30051-0308-000, 7 Amps, 250 VDC, 8 circuit), which was housed in an aluminum support (McMaster-Carr, machined in-house). R10-2RS bearings and bearing retainers (McMaster-Carr) were used to support the shaft at the slip-ring connection. A standpipe was used to flow reaction solution into the cell, machined in-house with PTFE tubing and elbows (McMaster-Carr). The entire reactor was supported with an 80/20 series 10 1" Aluminum frame (Grainger, machined in-house).

The inner electrode in the rotating cylinder reactor was a rod with 0.875" diameter and the outer electrode was a cylinder with 1.031" inner diameter. Both electrodes were 6" long. This resulted in 106 cm^2 surface area for the inner electrode and 125 cm^2 for the outer electrode.

Electrode Materials

In this report, we focus on using graphite, stainless steel, and zinc electrodes. However, the electrode materials in both reactors can be adapted to accommodate any solid material that can be machined into the specific geometry required for either the capillary gap or rotating cylinder reactor. Furthermore, electrodes can be coated using widely available physical and chemical coating techniques.[3–6] This means that there are a wide variety of electrode materials that can be fabricated and used in both reactors.

Electrode and Reactor Parts Cleaning Procedure

Solketal oxidation and XEC did not show signs of electrode fouling after runs, but electrodes were visibly fouled following the sulfonamide coupling reaction. Electrode cleaning was completed between every run to prevent contamination between experiments and to regenerate clean electrodes after fouling for the sulfonamide coupling reaction.

The procedure for cleaning electrodes was as follows:

1. Rinse with 1 M HCl. (Graphite and Steel only)
2. Rinse with acetone.
3. Polish with sandpaper of 50 grit, 100 grit, and then 220 grit.
4. Rinse with acetone.
5. Sonicate in acetone for 15 min. (Steel and Zinc only)
6. Dry at 80°C.

Teflon parts were cleaned by rinsing with 1 M HCl, rinsing with acetone, and then dried by wiping with WypAll Power Clean X80 Heavy Duty Cloths. Tubing was cleaned by rinsing with 1 M HCl, rinsing with acetone, and then blowing with an air hose to dry.

Flow Regimes and the Reynolds Number

Flow regimes in cells can be categorized and better understood through the use of the Reynolds number, Re. The Reynolds number is a dimensionless number that represents the ratio of the inertial forces to the viscous forces in a flowing fluid. [7]

$$\textbf{Equation S1:}\ \ \mathrm{Re} = \frac{\rho u L}{\mu}$$

The Reynolds number can be calculated from **Equation S1**, where ρ is the density of the fluid, u is the linear velocity of the fluid, and μ is the dynamic viscosity of the fluid. L is a characteristic length and depends on the geometry of the cell through which fluid is flowing. Additional variations to the Reynolds number are based on the geometry, with more variation from **Equation S1** with more complex geometries. When fluid is flowed radially between two circular plates, as in the capillary gap cell, the Reynolds number, denoted Re_{rad}, is described by **Equation S2**, where Q is the volumetric flow rate and r is the distance from the center of the circular plate.[8,9] In this geometry, the Reynolds number ranges from a maximum at the center of the circular plate to a minimum at the edge of the circular plate. When fluid is flowing axially in an annular region between two cylinders, as in the rotating cylinder cell, the Reynolds number, denoted Re_{RC}, incorporates the angular velocity Ω to calculate the linear velocity with L equivalent to $(R_o - R_i)$, where R_o is the radius of the outer cylinder and R_i is the radius of the inner cylinder.[10] Re_{RC} is given in **Equation S3**.

$$\textbf{Equation S2:}\ \ \mathrm{Re_{rad}} = \frac{\rho Q}{\pi \mu r}$$

$$\textbf{Equation S3:}\ \ \mathrm{Re_{RC}} = \frac{\rho \Omega R_i (R_o - R_i)}{\mu}$$

The Reynolds number can inform flow properties. The critical Reynolds number, Re_c, describes the Reynolds number below which laminar flow dominates and above which turbulent flow dominates. Laminar flow is characterized by fluid particles moving into the direction of flow without intermixing between layers of fluid. This flow regime fosters rigorously diffusive transport. In contrast, turbulent flow is characterized by a chaotic dispersion of fluid particles with considerable fluid movement in directions other than the principal flow. This flow regime promotes significant convective transport. Above the critical Reynolds number, the inertial forces overcome the viscous forces such that the laminae of fluid are disrupted, and chaotic dispersion of fluid particles ensues. In reality, more flow regimes exist beyond just laminar and turbulent flow, especially for complex flow geometries. The flow regimes associated with Taylor Couette flow, the flow present in the rotating cylinder cell, have been extensively discussed in previous studies.[10,11]

The critical Reynolds number for radial flow between circular plates, as in the capillary gap cell, is approximately 2000.[9] The critical Reynolds number for Taylor Couette flow, as in the rotating cylinder cell, is approximately 1250 for the dimensions of our reactor.[10] Reynolds numbers for the capillary gap and rotating cylinder cells are calculated for each reaction solution in **Table S1**. As can be seen, the capillary gap cell with 1 mm gaps rigorously promotes laminar flow while the rotating cylinderfosters turbulent flow when operating at 1000 RPM. The fluid properties used in the calculations are approximated from the solvents used for each chemistry and are given in **Table S2**.

Table S1. Estimated Flow Regimes in Flow Cells

Cell Type	Re_c	Reaction	Flow Rate (mL min^{-1})	Re	Flow Regime
Capillary gap (1 mm gaps)	2000	Solketal Oxidation	100	3 – 42	Laminar
		XEC	20	0.6 – 8.5	Laminar
		Sulfonamide Coupling	100	6 – 94	Laminar
Rotating cylinder (1000 RPM)	1250	Solketal Oxidation	100	2323	Turbulent
		XEC	20	2370	Turbulent
		Sulfonamide Coupling	100	5233	Turbulent

Table S2. Fluid Properties of Solvents Used in Reaction Solutions

Reaction	Density, ρ (g mL^{-1})	Dynamic viscosity, μ (cP)	Ref
Solketal Oxidation	0.998	1.0	9
XEC	0.937	0.92	10
Sulfonamide Coupling	0.787	0.35	11

Mass Transport Calculations and Computational Simulations

Particle Mean Transport Time Calculations

The mean transport time for diffusive transport in the capillary gap cell was approximated through application of the mean squared displacement, assuming diffusion of a Brownian particle in one dimension.[7,12] The mean displacement represents the average distance a particle diffuses from its original position, which is dependent on the time of diffusion. Therefore, when the mean displacement is assumed to be the distance between the center of the radial flow channel and the electrode surface, we can approximate the transport time for a particle to diffuse this distance. The calculation for diffusion time, t_{diff}, is given in **Equation S4**, where $<x^2>$ is the mean squared displacement with $x = 0.5$ mm (half of the interelectrode gap) and D is the diffusivity of the particle. D was approximated for solketal in water to be on the order of 10^{-5} cm^2 s^{-1} based on diffusivity reports for similar molecules.[13]

Equation S4: $$t_{diff} = \frac{<x^2>}{2D}$$

The mean transport time for a Tayor vortex flow regime was calculated based on the convective transport controlled by Taylor vortex formation. We can approximate the rotational speed of the vortices in the cell to be equivalent to the rotational speed of the internal cylinder, which allows calculation of the mean transport time for a particle in the center of the annular flow channel to the electrode surface based on the rotation rate of the internal cylinder. This calculation, which is only valid for Taylor vortex flow, is given by **Equation S5**, where x is the radial transport distance of 1 mm (half of the interelectrode gap), Ω is the angular velocity at 1000 RPM, R_i is the radius of the internal cylinder, and t_{conv} is the convective transport time. This calculation is not generalizable to other flow regimes that can be accessed in Taylor-Couette reactors.

Equation S5: $$t_{conv} = \frac{x}{\Omega Ri}$$

Computer Simulations

Computational fluid dynamic simulations were built using ANSYS Fluent 2020R2. Postprocessing of the simulations were completed using ANSYS Ensight 2022R2. Simulations of the capillary gap reactor were completed using exact electrode dimensions for two electrodes spaced 1 mm apart of the reactor using 25 mL min^{-1} flow rate. Simulations of the rotating cylinder reactor were completed using exact electrode dimensions for an internal rotation speed of 375 RPM. The fluid properties of solketal oxidation reaction shown in **Table S2** were used to complete these computational simulations.

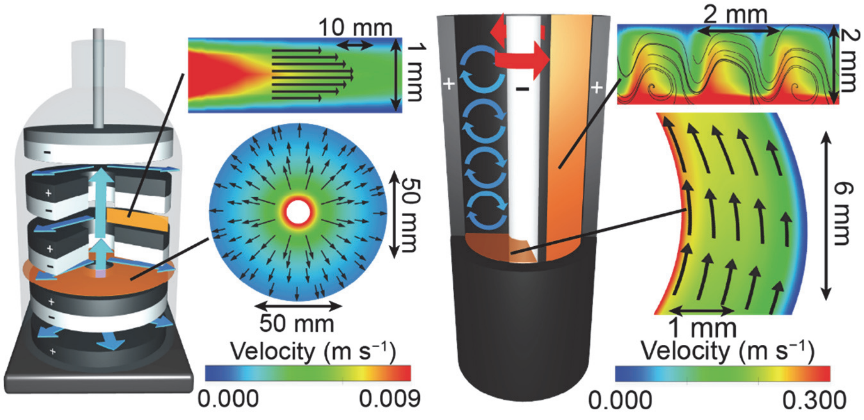

Figure S1. Images of CFD Simulations

Solketal Oxidation

Batch Potential Variation Experiments (Figure 2)

Solketal (2.5 mmol, 0.311 mL, 0.025 M) was solubilized in 100 mL MilliQ water in a beaker-type cell. To the stirred solution, ACT (0.125 mmol, 0.0268 g, 0.00125 M), $NaHCO_3$ (10 mmol, 0.840 g, 0.1 M), and Na_2CO_3 (10 mmol, 1.06 g, 0.1 M) were added. The solution was stirred until homogenous. Alternating graphite (7x) and stainless steel (7x) ¼" diameter rod electrodes were each submerged 2.36" into the solution, held in place by a Teflon guide on top of the cell (**Figure S2**). The interelectrode gap was 0.13". This batch cell was provided by AbbVie and has been used in previous reports.[14] The solution was stirred with a stir bar at 1100 RPM. The potential across the electrodes was held constant at 2.0 V or 3.0 V and the reaction was stopped once 4 F mol^{-1} was passed. 500 μL aliquots were taken before the reaction started and throughout the reaction and analyzed as below.[14]

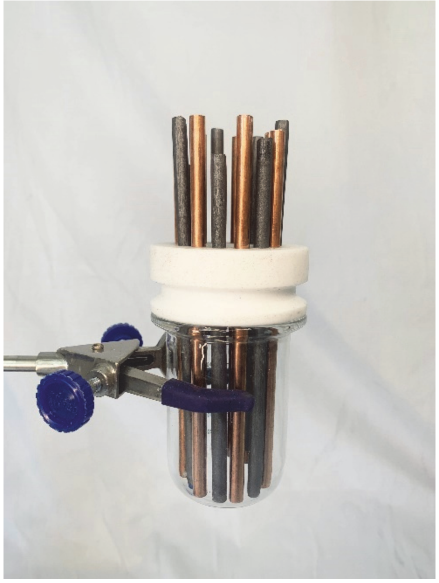

Figure S2: Batch cell used in preliminary screening of solketal oxidation. 7 stainless steel and 7 graphite rods were held in place by a Teflon guide.[14]

General Procedure – Capillary Gap Cell (Figure 6)

Solketal (10 mmol, 1.24 mL, 0.025 M) was solubilized in 400 mL MilliQ water. To the stirred solution, ACT (0.5 mmol, 0.107 g, 0.00125 M), $NaHCO_3$ (40 mmol, 3.36 g, 0.1 M), and Na_2CO_3 (40 mmol, 4.24 g, 0.1 M) were added. The solution was stirred until homogenous. Meanwhile, the capillary gap cell was assembled with graphite and stainless steel electrodes. Reaction solution was pumped into the cell at 100 mL min^{-1} and recirculated in a beaker outside of the cell. The potential across the entire cell was held constant at 8.0 V (2.0 V for each electrode pair) and the reaction was stopped after 12 h. 500 μL aliquots were taken before the reaction started and throughout the course of the reaction from the recirculation vial. NMR samples were prepared from the collected aliquots using D_2O as a solvent and DMSO as an external standard. 100 μL of aliquot and 50 μL of 0.0223 M DMSO in D_2O were added to 500 μL of D_2O. The ^{1}H-NMR spectra

used for characterization and quantification matched those from literature.[15] Solketal: δ 3.59 (dd, 1H), δ 3.69 (dd, 1H), δ 3.79 (dd, 1H); solketal carboxylate product: δ 3.93 (dd, 1H).

General Procedure – Rotating Cylinder Cell (Figure 6)

Solketal (2.5 mmol, 0.311 mL, 0.025 M) was solubilized in 100 mL MilliQ water. To the stirred solution, ACT (0.125 mmol, 0.0268 g, 0.00125 M), $NaHCO_3$ (10 mmol, 0.840 g, 0.1 M), and Na_2CO_3 (10 mmol, 1.06 g, 0.1 M) were added. The solution was stirred until homogenous. Meanwhile, the rotating cylinder cell was assembled with graphite outer electrode and stainless steel inner electrode. The inner electrode was set to rotate counterclockwise at 375 RPM. (This technically fosters modulated wavy vortex flow, which is still predominated by convective mixing).[10] Reaction solution was pumped into the cell at 100 mL min^{-1} and recirculated in a beaker outside of the cell. The potential across the cell was held constant at 2.0 V and the reaction was stopped after 4 F mol^{-1} was passed. 500 μL aliquots were taken before the reaction started and throughout the reaction from the recirculation vial and analyzed as above.

Current Densities for Runs in Recirculation

The current densities here correspond to the solketal oxidation runs in the capillary gap and rotating cylinder cells reported in **Figure 6** of the main text.

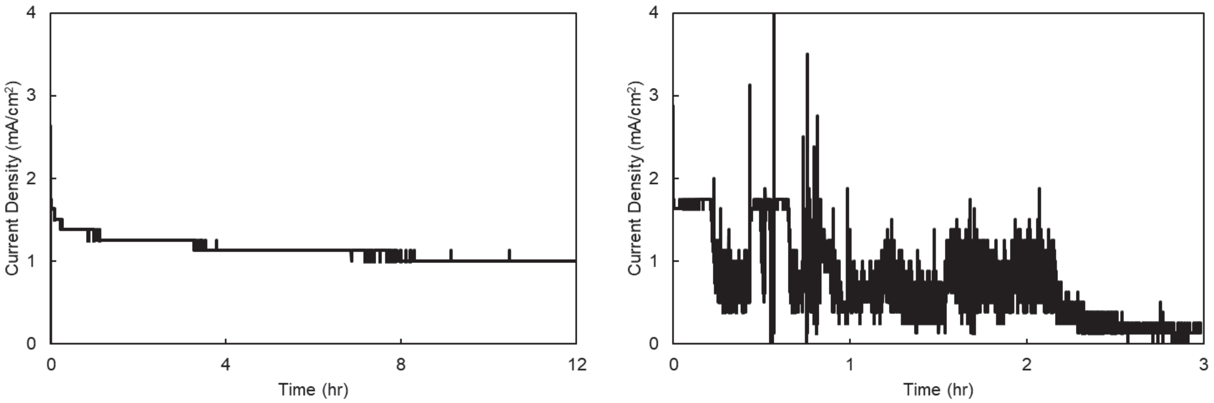

Figure S3. Current densities for solketal oxidation (Figure 6, Reaction 1) in the capillary gap (left) and rotating cylinder (right) cells.

Runs in Recirculation at Varying Flow Rates

The results reported here follow the same procedure for solketal oxidation as described above but the fluid is pumped at the indicated flow rate.

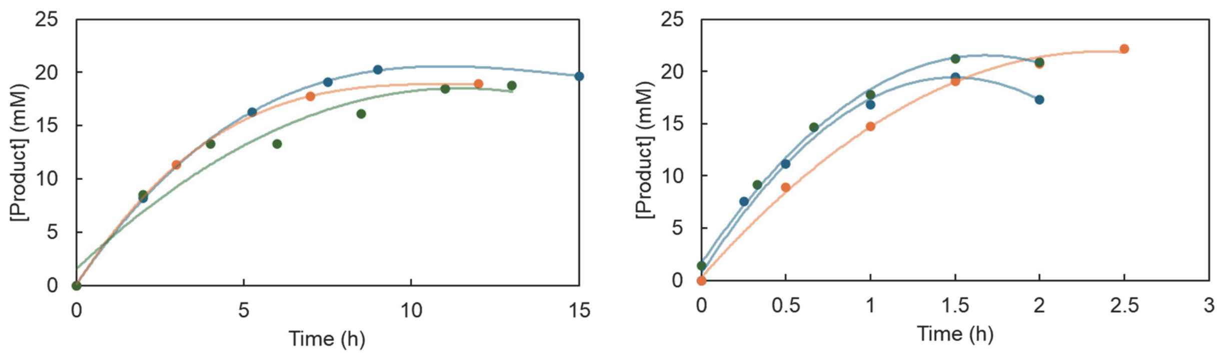

Figure S4. Solketal oxidation (Figure 6, Reaction 1) in the capillary gap (left) and rotating cylinder (right) cells at varying flow rates. (Blue = 500 mL min^{-1}, Orange = 100 mL min^{-1}, Green = 20 mL min^{-1}). Lines serve as guides to the eye.

General Procedure – Rotating Cylinder Cell at Single-Pass (Figure 7)

Solketal (100 mmol, 12.4 mL, 0.5 M) was solubilized in MilliQ water to total 200 mL. To the stirred solution, ACT (5 mmol, 1.07 g, 0.025 M), $NaHCO_3$ (100 mmol, 8.40 g, 0.5 M), and Na_2CO_3 (100 mmol, 10.6 g, 0.5 M) were added. The solution was stirred until homogenous. Meanwhile, the rotating cylinder cell was assembled with graphite outer electrode and stainless steel inner electrode. The inner electrode was set to rotate counterclockwise at 1500 RPM. Reaction solution was pumped into the cell at 1 mL min^{-1} without recirculation and a flow meter (Bronkhorst, mini CORI-FLOW M12) was used to monitor the flow rate. The potential across the cell was held constant at 3.0 V and the reaction was stopped after 144 min (approximately 144 mL of reaction solution, or 72 mmol substrate). 500 µL aliquots were taken before the reaction started and throughout the reaction directly from the outlet of the reactor and analyzed as above.

Cross-Electrophile Coupling

Batch Potential Variation Experiments (Figure 3)

To an undivided 5 mL custom-made electrochemical cell (**Figure S5**) $NiBr_2$ trihydrate (0.065 mmol, 17.7 mg, 5 mol%), 4,4’-dimethoxy-2,2’-bipyridine (0.065 mmol, 14.1 mg, 5 mol%), 1,3,5-trimethoxybenzene (internal standard, 0.43 mmol, 72.9 mg), and a cross-shaped stir bar were added. The cell was then transferred into a N_2-filled glovebox where NaI (0.2 M, 1.00 mmol, 155 mg), ethyl 4-bromobenzoate (1.3 mmol, 0.21 mL, 0.25 M), 3-phenylpropyl bromide (1.7 mmol, 0.26 mL, 0.33 M), and 5 mL of anhydrous DMA were added. Once the reaction mixture was fully homogenized, the cell was sealed with a custom lid bearing the two electrodes (graphite rod and zinc plate) and clamped. The cell was brought out of the glovebox and connected to N_2 through a Teflon cannula. The two electrodes, graphite rod (cathode, diameter: 0.15 cm) and Zn rod (anode, diameter: 0.15 cm) were each submerged 2 cm into solution. The reaction was run under constant applied cell potential of −1 V or −2 V. The reaction was stopped once 2.1 F mol^{-1} were passed. 100 µL aliquots were taken at the end of electrolysis, diluted with 500 µL of $CDCl_3$ and analyzed throughout ^{1}H-NMR spectroscopy using 1,3,5-trimethoxybenzene as internal standard. Analysis by NMR was performed as below.

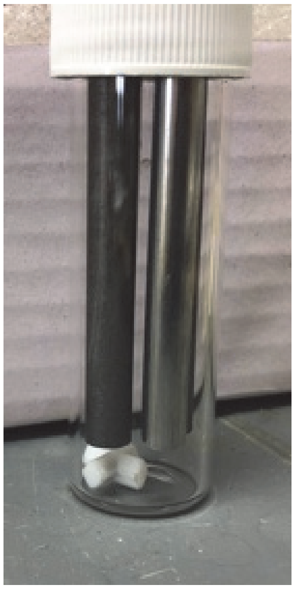

Figure S5: Batch cell used in preliminary cross-electrophile coupling experiments.

General Procedure – Capillary Gap Cell (Figure 6)

The following procedure was performed in a nitrogen-filled purge box. To 400 mL of DMA stirring on a stir plate, $NiBr_2$ trihydrate (5 mmol, 1.36 g, 0.0125 M), 4,4’-dimethoxy-2,2’-bipyridine (5 mmol, 1.08 g, 0.0125 M), and NaI (80 mmol, 12.0 g, 0.2 M) were added and stirred until the solution turned dark green and all solids were dissolved. Ethyl 4-bromobenzoate (100 mmol, 16.3 mL, 0.25 M) and 3-phenylpropyl bromide (132 mmol, 20.1 mL, 0.33 M) were then added. Meanwhile, the capillary gap cell was assembled with graphite and zinc electrodes. Reaction solution was pumped into the cell at 20 mL min^{-1} and recirculated in a beaker outside of the cell. The potential across the entire cell was held constant at either −2 V (−0.5 V per electrode pair) or −4 V (−1 V per electrode pair) and the reaction was stopped once

2 F mol^{-1} was passed. 500 μL aliquots were taken before the reaction started and throughout the course of the reaction from the recirculation vial. NMR samples were prepared from the collected aliquots using $CDCl_3$ as a solvent and 1,3,5-trimethoxybenzene (TMB) as an external standard. 100 μL of aliquot and 50 μL of 0.133 M TMB in $CDCl_3$ were added to 700 μL of $CDCl_3$. The solution rested until a precipitate formed and 600 μL of the solution (without precipitate) was taken for NMR analysis. The ^{1}H-NMR spectra used for characterization matched those from literature.[16] Ar–Br substrate: δ 7.58 (d, 2H), δ 7.91 (d, 2H); Ar–Alkyl product: δ 7.96 (d, 2H); Ar–Ar: δ 8.14 (d, 2H); Ar–H: δ 8.05 (d, 2H).

General Procedure – Rotating Cylinder Cell (Figure 6)

The following procedure was performed entirely in a nitrogen-filled glovebox. To 100 mL of DMA stirring on a stir plate, $NiBr_2$ trihydrate (1.25 mmol, 0.34 g, 0.0125 M), 4,4'-dimethoxy-2,2'-bipyridine (1.25 mmol, 0.27 g, 0.0125 M), and NaI (20 mmol, 3.0 g, 0.2 M) were added and stirred until the solution turned dark green and all solids were dissolved. Ethyl 4-bromobenzoate (25 mmol, 4.08 mL, 0.25 M) and 3-phenylpropyl bromide (33 mmol, 5.02 mL, 0.33 M) were then added. Meanwhile, the rotating cylinder cell was assembled with graphite inner electrode and zinc outer electrode. The inner electrode was set to rotate counterclockwise at 1000 RPM. Reaction solution was pumped into the cell at 20 mL min^{-1} and recirculated in a beaker outside of the cell. The potential across the cell was held constant at either −0.5 V or −1 V and the reaction was stopped once 2 F mol^{-1} had been passed. 500 μL aliquots were taken before the reaction started and throughout the course of the reaction from the recirculation vial and analyzed as above.

Current Densities for Runs in Recirculation

The current densities here correspond to the XEC runs in the capillary gap and rotating cylinder cells reported in **Figure 6** of the main text.

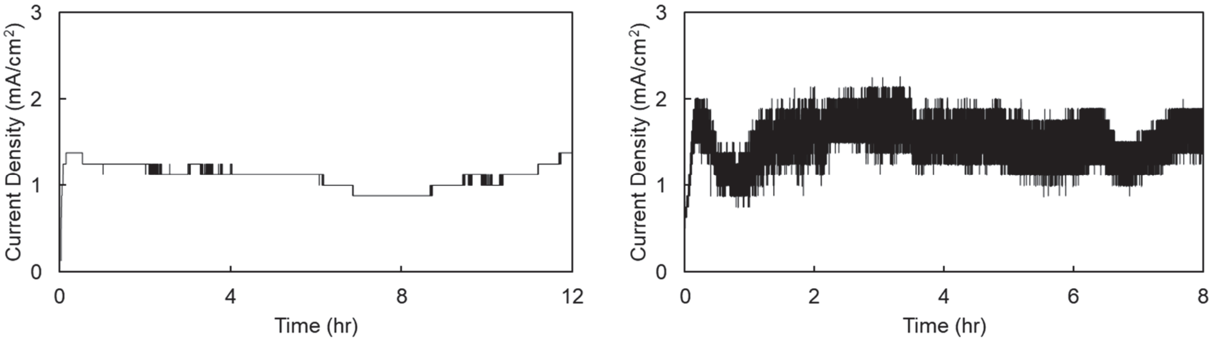

Figure S6. Current densities for XEC at −0.5 V (Figure 6, Reaction 2) in the capillary gap (left) and rotating cylinder (right) cells.

Data for XEC Performed in Recirculation at −1.0 V

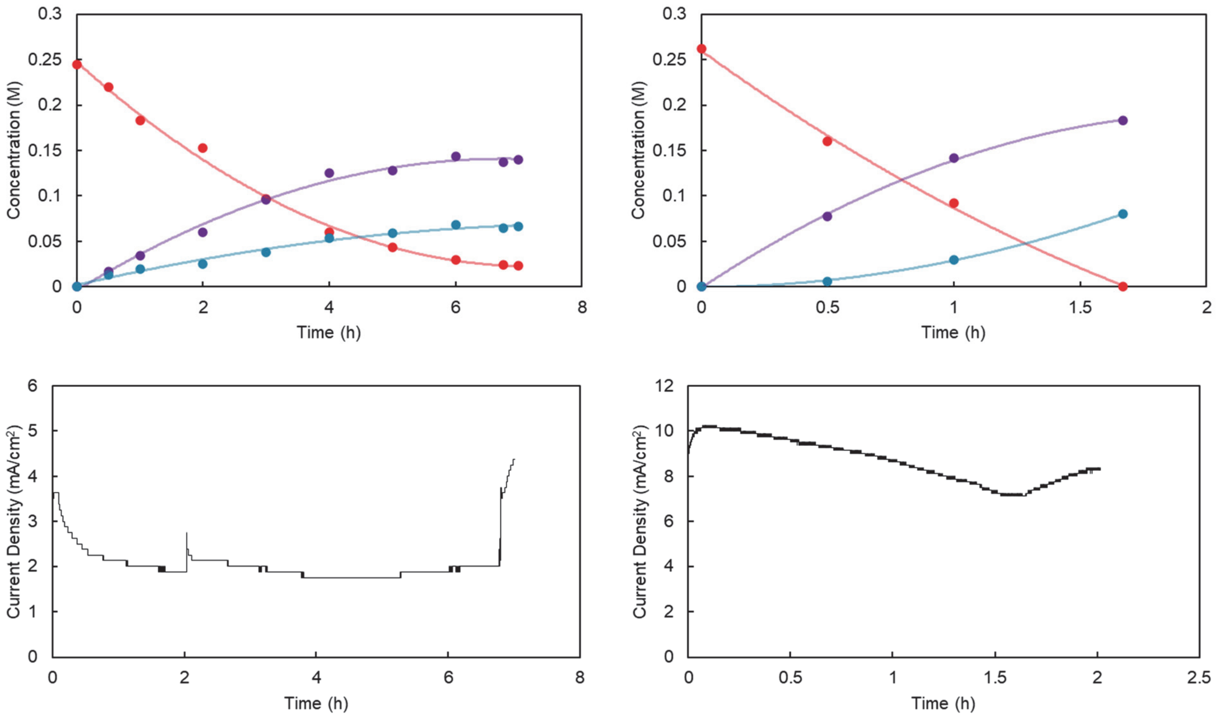

Figure S7. Reaction time courses (top) and current densities (bottom) for XEC at −1.0 V in the capillary gap (left) and rotating cylinder (right) cells. Red = Ar–Br; Purple = product, Blue = sum of byproducts (Ar–H and Ar–Ar). Lines serve as guides to the eye.

General Procedure – Rotating Cylinder Cell at Single-Pass (Figure 7)

The following procedure was performed entirely in a nitrogen-filled glovebox. 200 mL of DMA was stirred on a stir plate, $NiBr_2$ trihydrate (1.25 mmol, 0.34 g, 0.00625 M), 4,4’-dimethoxy-2,2’-bipyridine (1.25 mmol, 0.27 g, 0.00625 M), and NaI (40 mmol, 6.0 g, 0.2 M) were added and stirred until the solution turned dark green and all solids were dissolved. Ethyl 4-bromobenzoate (25 mmol, 4.08 mL, 0.125 M) and 3-phenylpropyl bromide (33 mmol, 5.02 mL, 0.165 M) were then added. Meanwhile, the rotating cylinder cell was assembled with graphite inner electrode and zinc outer electrode. The inner electrode was set to rotate counterclockwise at 1500 RPM. Reaction solution was pumped into the cell at 0.5 mL min^{-1} without recirculation. A flow meter (Bronkhorst, mini CORI-FLOW M12) was used to monitor the flow rate. The potential across the cell was held constant at –0.5 V and the reaction was stopped after 6 h (corresponding to approximately 180 mL and 22.5 mmol substrate). 500 μL aliquots were taken before the reaction started and throughout the course of the reaction directly from the outlet and analyzed as above.

SEM Imaging for XEC Reaction

Scanning electron microscopy (SEM) with EDX analysis was completed on graphite electrodes used in the capillary gap reactor before and after completing a Ni-catalyzed XEC reaction (general procedure in recirculation as described above) to evaluate the elemental

composition of the electrode surface. The SEM images and EDX analysis are given in **Figure S8** and **S9**. We see no significant changes in the EDX analysis before and after the reaction, suggesting negligible modification of the elemental composition of the electrode during the reaction.

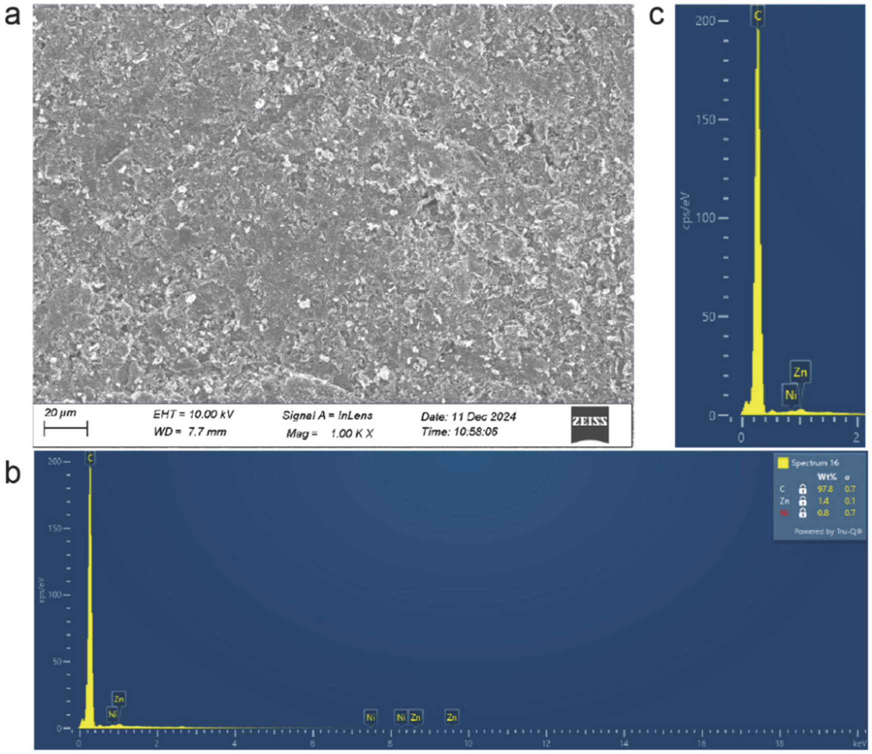

Figure S8: (a) SEM image and **(b)** EDX analysis of graphite plate from capillary gap reactor before Ni-catalyzed XEC reaction. **(c)** Magnified EDX spectrum.

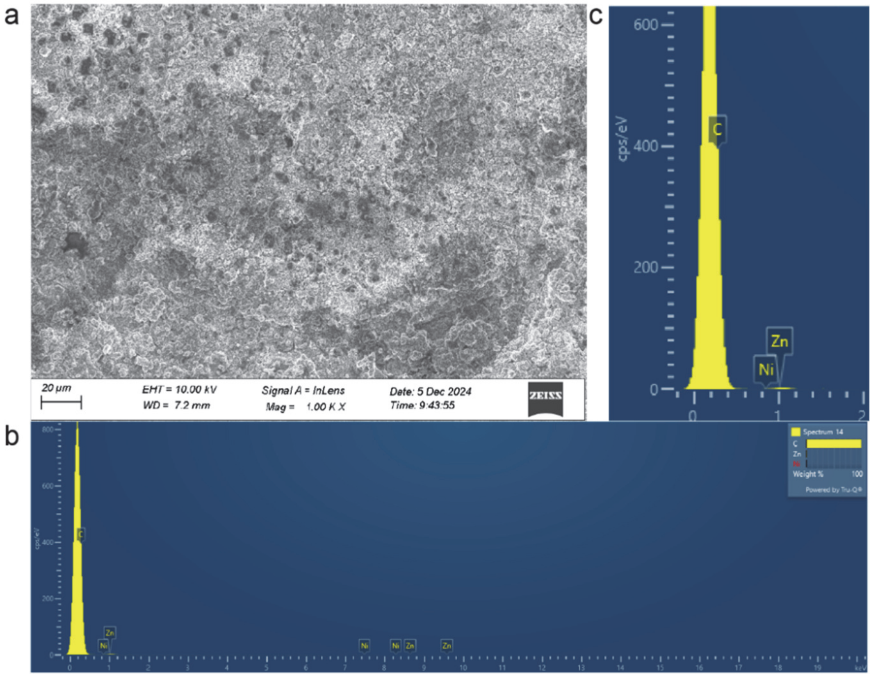

Figure S9: (a) SEM image and **(b)** EDX analysis of graphite plate from capillary gap reactor after Ni-catalyzed XEC reaction. **(c)** Magnified EDX spectrum.

Sulfonamide Coupling

Parallel Plate Acid Concentration Variation Experiments (Figure 4)

7.5 mL of 1 M HCl was added to 92.5 mL of acetonitrile. While the solution was stirred on a stir plate and purged with N_2, 4,6-dimethyl-2-mercaptopyrimidine (10 mmol, 1.40 g, 0.1 M), cyclohexylamine (15 mmol, 1.72 mL, 0.15 M), and tetrabutylammonium tetrafluoroborate (1 mmol, 0.33 g, 0.01 M) were added and dissolved. Meanwhile, a parallel plate cell with internal cell dimensions of 9.2 cm by 8.0 cm and 0.57 cm interelectrode gap (**Figure S10**) was assembled with graphite and stainless steel plate electrodes. Reaction solution was pumped into the cell at 100 mL min^{-1} and recirculated in a beaker outside of the cell. The potential across the entire cell was held constant at 3.4 V and the reaction was stopped once 9 F mol^{-1} was passed. 500 µL aliquots were taken before the reaction started and throughout the reaction from the recirculation vial and analyzed as below.

General Procedure – Capillary Gap Cell (Figure 6)

30 mL of 1 M HCl was added to 370 mL of acetonitrile. While the solution was stirred on a stir plate and purged with N_2, 4-6-dimethyl-2-mercaptopyrimidine (40 mmol, 5.61 g, 0.1 M), cyclohexylamine (60 mmol, 6.88 mL, 0.15 M), and tetrabutylammonium tetrafluoroborate (4 mmol, 1.32 g, 0.01 M) were added and dissolved. Meanwhile, the capillary gap cell was assembled with graphite and stainless steel electrodes. Reaction solution was pumped into the cell at 100 mL min^{-1} and recirculated in a beaker outside of the cell. The potential across the entire cell was held constant at 13.6 V (3.4 V for each electrode pair) and the reaction was stopped once 10 F mol^{-1} was passed. 500 µL aliquots were taken before the reaction started and throughout the reaction from the recirculation vial. NMR samples were prepared from the aliquots using CD_3CN as a solvent and 1,3,5-trimethoxybenzene (TMB) as an external standard. 50 µL of the collected aliquot were added to an Eppendorf tube with 18.75 µL of 0.2 M $NaHCO_3$ (to neutralize the 1 M HCl cosolvent) and solvent was removed under vacuum. The remaining solid was dissolved in 600 µL CD_3CN and 10 µL of 0.167 M TMB in CD_3CN was added for NMR analysis. The ^{1}H-NMR spectra used for characterization matched those from literature.[17] Substrate thiol: δ 6.50 (s, 1H); sulfonamide product: δ 7.31 (s, 1H); primary sulfonamide: δ 7.13 (s, 1H).

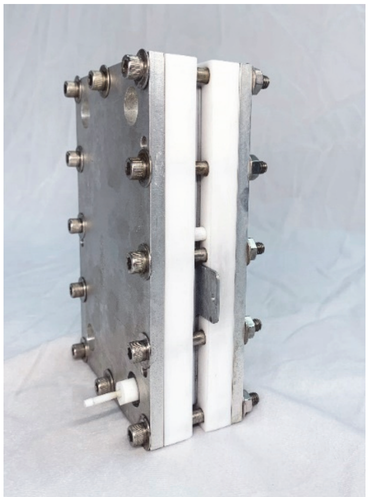

Figure S10: Parallel plate reactor used in preliminary sulfonamide coupling experiments.

General Procedure – Rotating Cylinder Cell (Figure 6)

7.5 mL of 1 M HCl was added to 92.5 mL of acetonitrile. While the solution was stirred on a stir plate and purged with N_2, 4-6-dimethyl-2-mercaptopyrimidine (10 mmol, 1.40 g, 0.1 M), cyclohexylamine (15 mmol, 1.72 mL, 0.15 M), and tetrabutylammonium tetrafluoroborate (1 mmol, 0.33 g, 0.01 M) were added and dissolved. Meanwhile, the rotating cylinder cell was assembled with graphite outer electrode and stainless steel inner electrode. The inner electrode was set to rotate counterclockwise at 1000 RPM. Reaction solution was pumped into the cell at 100 mL min^{-1} and recirculated in a beaker outside of the cell. The potential across the entire cell was held constant at 3.4 V and the reaction was stopped once 10 F mol^{-1} was passed. 500 μL aliquots were taken before the reaction started and throughout the reaction from the recirculation vial and analyzed as above.

Sulfonamide coupling was also completed in the rotating cylinder reactor with no rotation. The procedure is the same as described above but the motor was not turned on (effectively setting the rotation of the inner cylinder to 0 RPM) and the reaction was run until 10 F mol^{-1} had been passed.

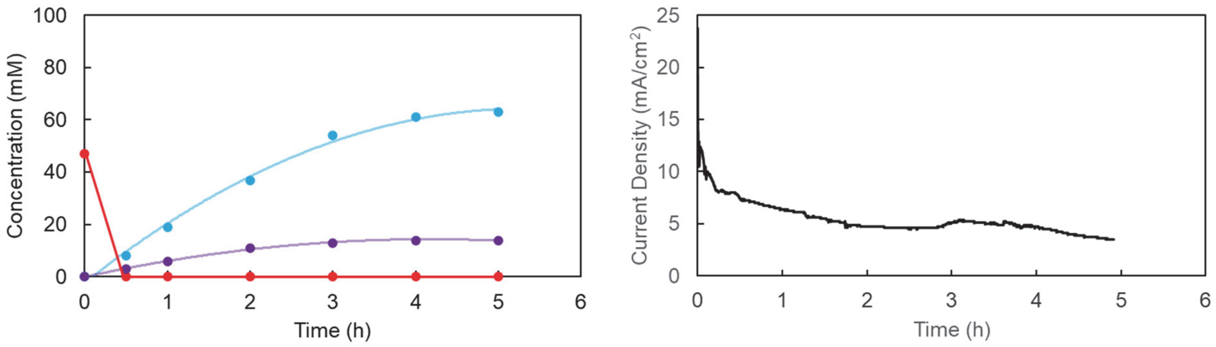

Figure S11. Performance of sulfonamide coupling in the rotating cylinder reactor at 0 RPM. Red = thiol, blue = sulfonamide product, purple = primary sulfonamide byproduct. Lines serve as guides to the eye.

Current Densities for Runs in Recirculation

The current densities here correspond to the sulfonamide runs in the capillary gap and rotating cylinder cells reported in **Figure 6** of the main text.

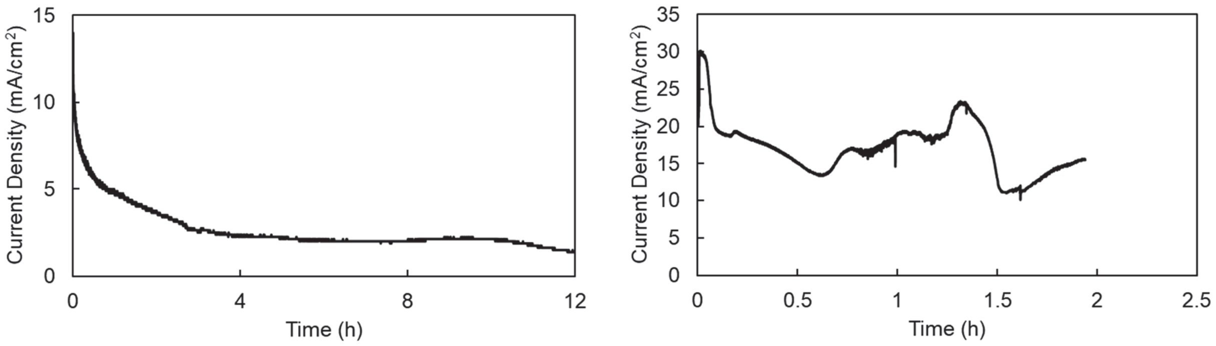

Figure S12. Current densities for sulfonamide coupling (Figure 6, Reaction 3) in the capillary gap (left) and rotating cylinder (right) cells.

General Procedure – Capillary Gap Cell in Single-Pass (Figure 7)

37.5 mL of 1 M HCl was added to 462.5 mL of acetonitrile. While the solution was stirred on a stir plate and purged with N_2, 4-6-dimethyl-2-mercaptopyrimidine (50 mmol, 7.01 g, 0.1 M), cyclohexylamine (75 mmol, 8.60 mL, 0.15 M), and tetrabutylammonium tetrafluoroborate (5 mmol, 1.65 g, 0.01 M) were added and dissolved. Meanwhile, the capillary gap cell was assembled with graphite and stainless steel electrodes. Reaction solution was pumped into the cell at 1.5 mL min^{-1} without recirculation. A flow meter (Bronkhorst, mini CORI-FLOW M12) was used to monitor the flow rate. The current across the entire cell was held constant at 7.3 mA cm^{-2} and the reaction was stopped after 270 min (corresponding to approximately 400 mL of reaction solution, or 41 mmol substrate). 500 µL aliquots were taken before the reaction started and during the course of the reaction directly from the outlet and analyzed as above.

Primary Sulfonamide Side Product Formation

Possible mechanisms for primary sulfonamide formation are given in **Figure S13.**

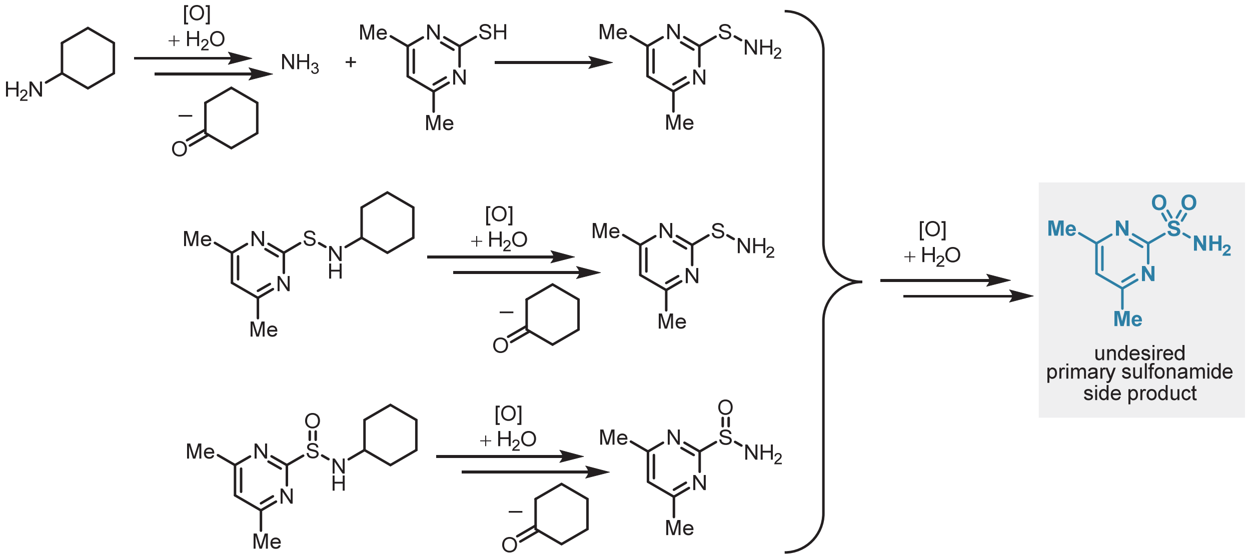

Figure S13: Three possible mechanisms of amine dealkylation that would lead to the primary sulfonamide side product formation in the sulfonamide coupling reaction.

Water Oxidation for Oxygen Supply Quantification

We investigated water oxidation to O_2 to investigate if sulfonamide coupling is limited by interfacial water availability. For this reaction, 37.5 mL of 1 M HCl was added to 462.5 mL of acetonitrile. While the solution was stirred on a stir plate, tetrabutylammonium tetrafluoroborate (5 mmol, 1.65 g, 0.01 M) was added and dissolved. Notably, no substrates were added. Meanwhile, the capillary gap reactor was assembled with one graphite and one stainless steel electrode and a leakless miniature Ag/AgCl reference electrode was set up at the inlet of the reactor. The graphite plate was used as the working electrode, which is the same as used for the sulfonamide coupling reaction. Reaction solution was flowed into the cell at 100 mL min^{-1} and recirculated through a beaker outside of the cell. The potential was swept from 0.65 V *vs* Ag/AgCl to 2.0 V *vs* Ag/AgCl, and the resulting current is shown in **Figure S14**. From this potential sweep,

we see the onset of water oxidation at 1.4 V *vs* Ag/AgCl and we see that no mass transport limited regime is reached even above 1.0 A, corresponding to an oxygen atom transfer rate far above that required for sulfonamide coupling under the experimentally observed current. The results show that water availability at the electrode surface is likely not limiting the sulfonamide coupling reaction.

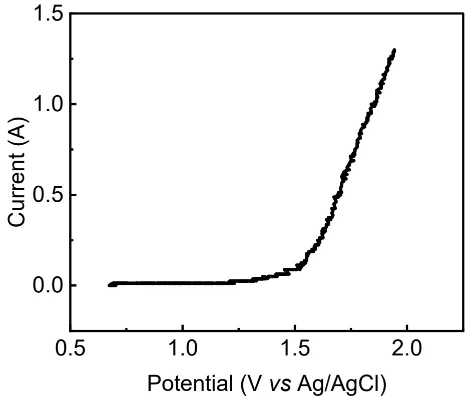

Figure S14: Linear sweep voltammogram of water oxidation in sulfonamide coupling reaction solution in the absence of substrate.

Electrode Characterization

We performed a series of electrode characterization measurements for all three reactions in both the capillary gap and rotating cylinder reactors. The electrochemical surface area (ECSA) was measured through cyclic voltammetry (CV) experiments and charge transfer resistances were measured using electrochemical impedance spectroscopy (EIS). We used a leakless miniature Ag/AgCl reference electrode (eDAQ, ET072-3) throughout these experiments, which was positioned at the inlet of the reactors. All electrode characterization experiments were carried out using a Gamry Interface 1010E potentiostat unless otherwise noted.

Each test was completed both before and after a reaction. All reactions were performed in recirculation under the conditions which were used for **Figure 6**. The general procedures for each of these reactions are described above.

Electrochemical Surface Area (ECSA)

We calculated the ECSA of the graphite working electrodes by measuring the double layer capacitance, C_{DL}, of the electrodes through CV experiments and by comparison to a measured specific capacitance, C_S.[18–21] This relation is given in **Equation S6**.

Equation S6: $$\mathrm{ECSA} = \frac{C_{DL}}{C_S}$$

The C_{DL} can be measured by performing CV experiments at a range of scan rates while recording the capacitive currents. The capacitive current, i_C, is directly proportional to the scan rate, ν, and when plotted as a function of scan rate, C_{DL} can be extracted, as shown in **Equation S7**.

Equation S7: $$i_C = C_{DL}\nu$$

CVs were recorded between 0.3 and 0.7 V *vs* Ag/AgCl in N_2-purged 0.15 M tetrabutylammonium tetrafluoroborate (TBA BF_4) in DMF at 0.5, 0.75, 1, 1.5, and 2 mV s^{-1}. Capacitive currents were calculated by taking half of the difference between the anodic and cathodic currents, i_a and i_c, respectively, at 0.5 V *vs* Ag/AgCl(**Equation S8**).

Equation S8: $$i_C = \frac{i_a - i_c}{2}$$

The C_S was determined by performing similar CV experiments to measure the C_{DL} of a glassy carbon stub in the same electrolyte (0.15 M TBA BF_4 in DMF). CVs were completed in a five-neck flask using an Autolab PGSTAT302N in the same potential range and scan rates as described above. The working electrode was a polished glassy carbon stub, the counter electrode was a Pt wire, and the reference electrode was a leakless Ag/AgCl electrode. In these measurements, we approximated C_S by making the assumption that the surface roughness of the polished glassy carbon stub was 1, meaning that the ECSA is equivalent to the geometric surface area (0.0707 cm^2).These results are shown in **Figure S15** and **Table S3**.

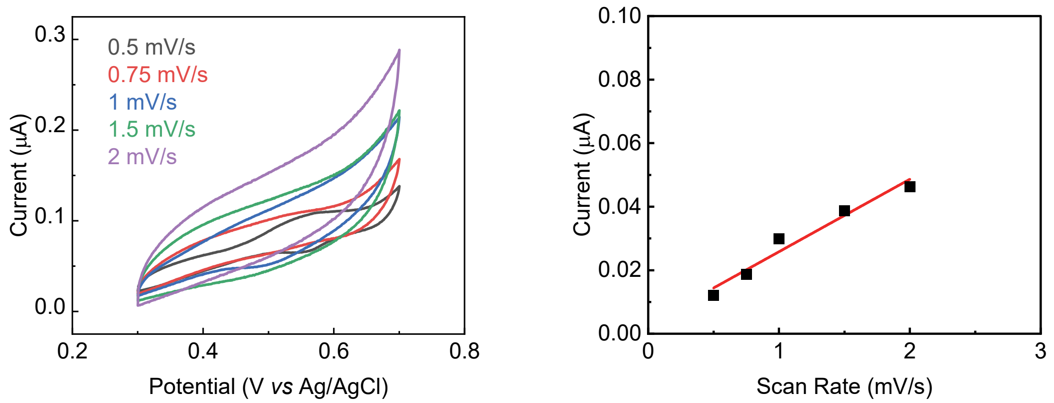

Figure S15: CVs and scan rate dependence for ECSA measurements of the polished glassy carbon electrode.

Table S3: Calculated C_{DL} of Glassy Carbon Stub

Reactor	Geometric Surface Area (cm^2)	Calculated C_{DL} (μF)	Specific Capacitance, C_S ($\mu F\ cm^{-2}$)
Capillary Gap	0.0707	22.8	322

We found a specific capacitance of 322 $\mu F\ cm^{-2}$ for the glassy carbon stub. This fits in the typical range for graphite and other carbon electrodes that have been previously reported for other electrolytes.[19,20]

We first determined the initial ECSA of the clean graphite working electrodes in both reactors. For these experiments, the capillary gap reactor was assembled with only one anode and one cathode. For the rotating cylinder reactor, both the graphite outer electrode (used for alcohol oxidation and sulfonamide coupling) and the graphite inner electrode (used for XEC) were measured using a stainless steel counter electrode. 0.15 M TBA BF_4 in DMF was flowed through the reactors at 100 mL min^{-1} and CV measurements were recorded as described above. The plots of current as a function of scan rate are shown in **Figure S16-S18** and the calculated ECSAs are shown in **Table S4**.

We then measured ECSAs of the graphite working electrodes after the reactions. Once the reactions were complete, the reactors were first completely drained of the reaction solution. For the capillary gap reactor, the reactor was partially disassembled, and three sets of anodes and cathodes were removed to leave only one anode and one cathode in the reactor for the ECSA measurement. The capillary gap reactor was then reassembled. For XEC reactions, the zinc electrode was replaced with a stainless steel electrode. Then, for both reactors, DMF was flowed through the reactor at 100 mL min^{-1} to remove any remaining solvent, electrolyte and substrate. The DMF was the drained, and the 0.15 M TBA BF_4 in DMF electrolyte was flowed through the reactor at 100 mL min^{-1}. CV measurements were subsequently recorded as described above. After the XEC reaction, the potential range between 0.3 and 0.7 V vs Ag/AgCl showed clear signs of Faradaic processes, so the potential window for ECSA characterization was lowered. In the capillary gap reactor after XEC, CVs were run from −0.6 to −0.2 V vs Ag/AgCl and capacitive currents were taken at −0.4 V vs Ag/AgCl. In the rotating cylinder reactor after XEC, CVs were run from −0.2 to 0.2 V vs Ag/AgCl and capacitive currents were taken at 0 V vs Ag/AgCl. The

plots of current as a function of scan rate are shown in **Figures S19-S24** and the calculated ECSAs are shown in **Table S5**.[21,22]

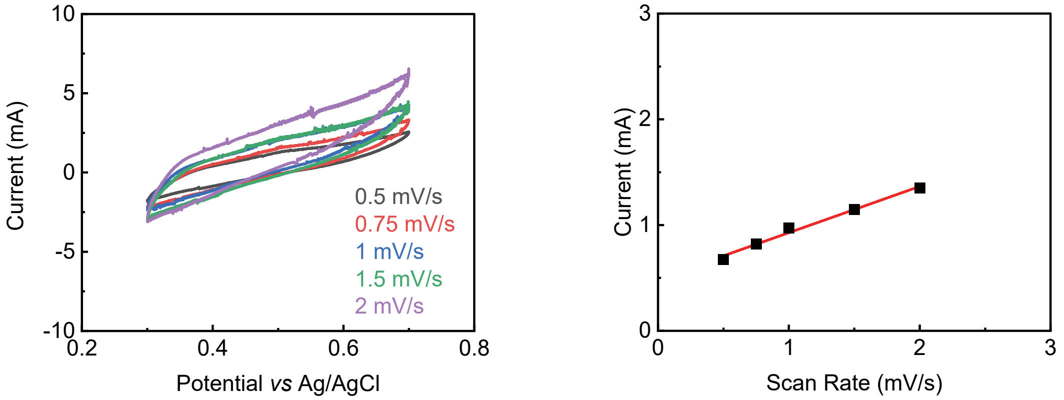

Figure S16: CVs (left) and scan rate dependence (right) for ECSA measurements of the clean graphite electrodes in the capillary gap reactor.

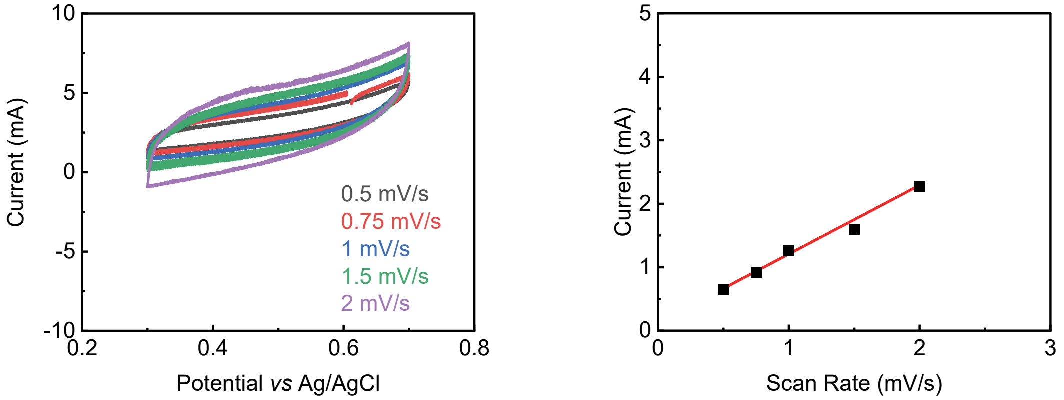

Figure S17: CVs (left) and scan rate dependence (right) for ECSA measurements of the clean graphite outer electrode in the rotating cylinder reactor. This electrode is used for alcohol oxidation and sulfonamide coupling.

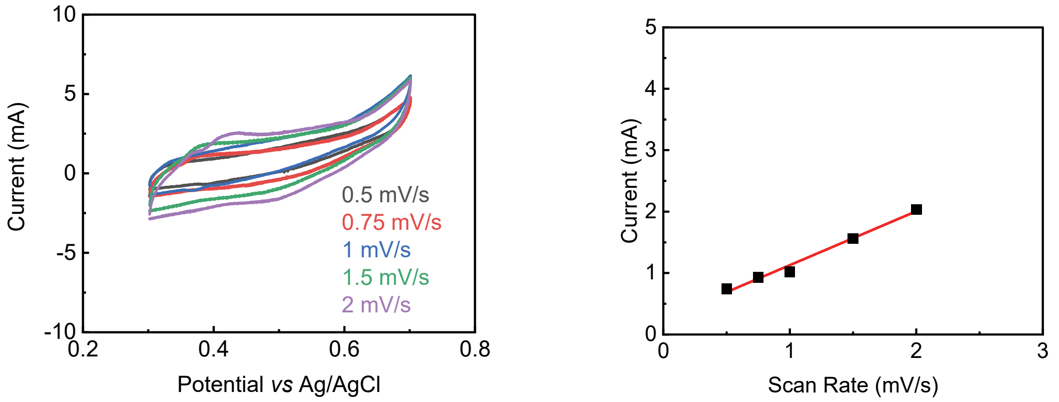

Figure S18: CVs (left) and scan rate dependence (right) for ECSA measurements of the clean graphite inner electrode in the rotating cylinder reactor. This electrode is used for Ni-catalyzed XEC.

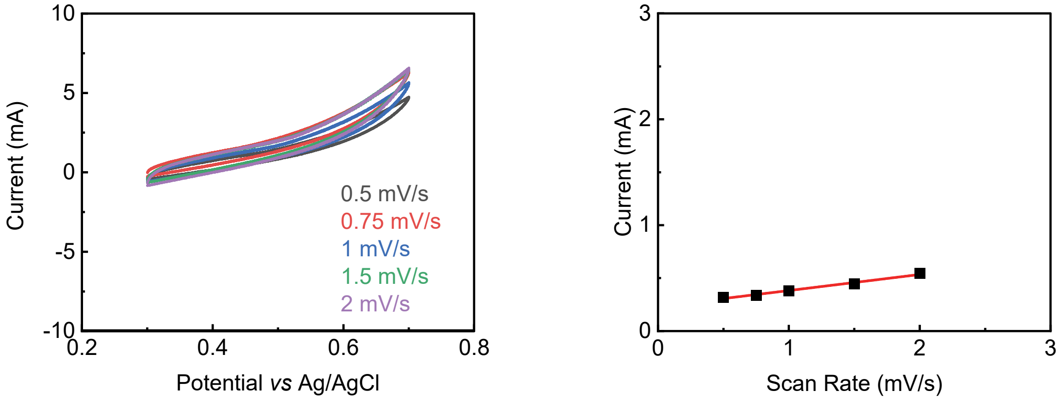

Figure S19: CVs (left) and scan rate dependence (right) for ECSA measurements of the graphite electrode in the capillary gap reactor after alcohol oxidation.

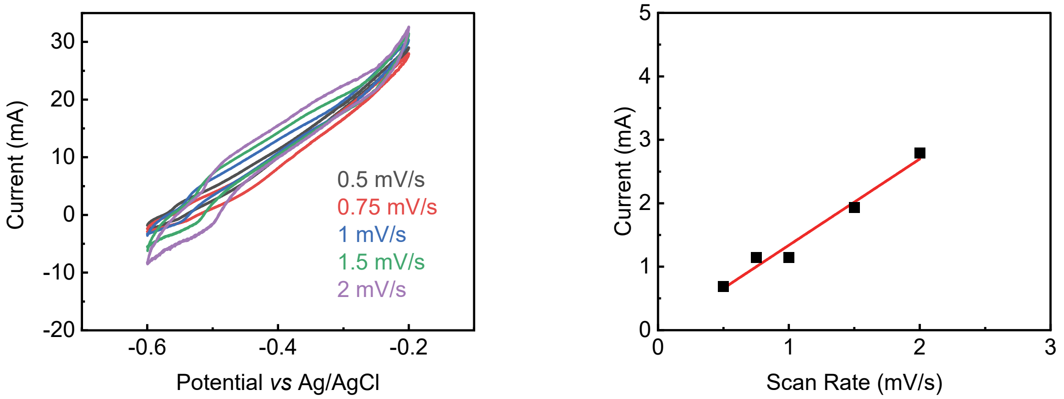

Figure S20: CVs (left) and scan rate dependence (right) for ECSA measurements of the graphite electrode in the capillary gap reactor after XEC.

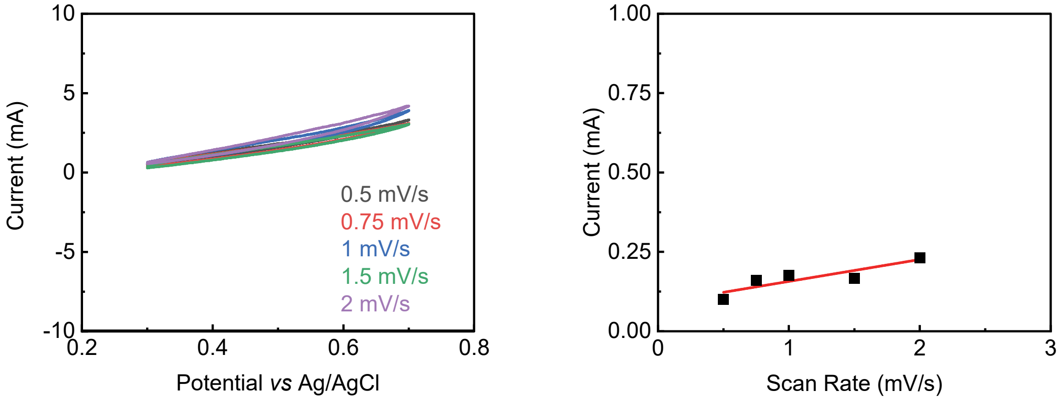

Figure S21: CVs (left) and scan rate dependence (right) for ECSA measurements of the graphite electrode in the capillary gap reactor after sulfonamide coupling.

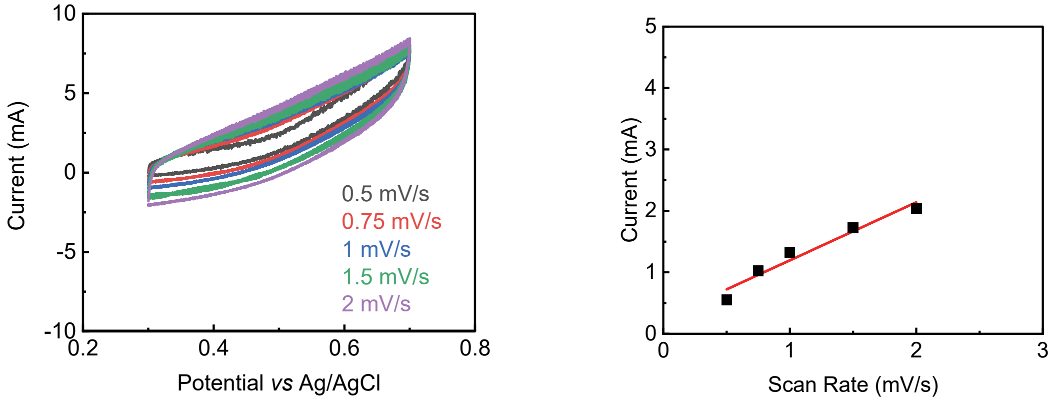

Figure S22: CVs (left) and scan rate dependence (right) for ECSA measurements of the graphite outer electrode in the rotating cylinder reactor after alcohol oxidation.

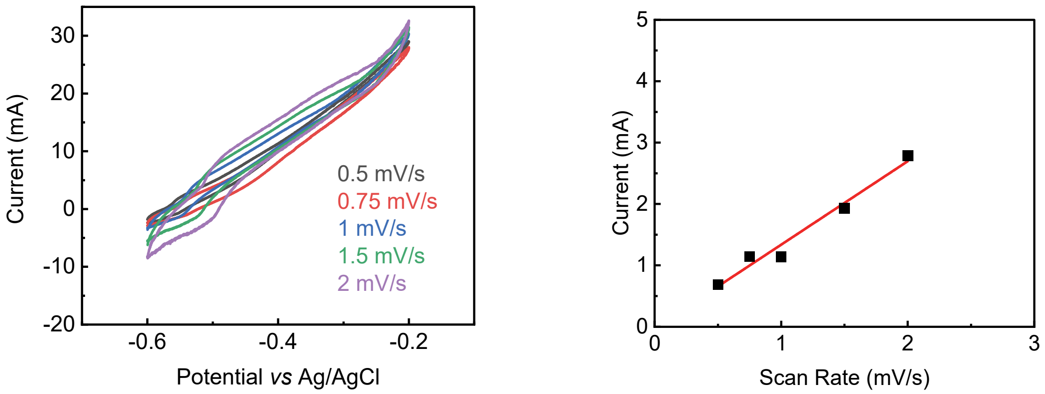

Figure S23: CVs (left) and scan rate dependence (right) for ECSA measurements of the graphite inner electrode in the rotating cylinder reactor after Ni-catalyzed XEC.

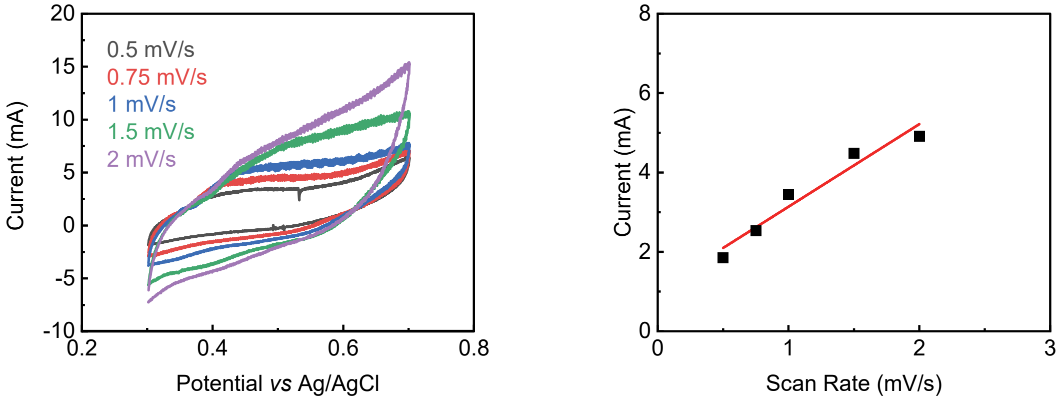

Figure S24: CVs (left) and scan rate dependence (right) for ECSA measurements of the graphite outer electrode in the rotating cylinder reactor after sulfonamide coupling.

Table S4: Calculated C_{DL} for Graphite Working Electrodes in Capillary Gap and Rotating Cylinder Reactors

Reactor	C_{DL} Before Reaction (mF)	C_{DL} After Alcohol Oxidation (mF)	C_{DL} After XEC (mF)	C_{DL} After Sulfonamide Coupling (mF)
Capillary Gap	438	152	1359	68.9
Rotating Cylinder (outer electrode)	1049	946	N/A	2081
Rotating Cylinder (inner electrode)	878	N/A	142	N/A

Table S5: Calculated ECSA for Graphite Working Electrodes in Capillary Gap and Rotating Cylinder Reactors

Reactor	ECSA Before Reaction (cm^2)	ECSA After Alcohol Oxidation (cm^2)	ECSA After XEC (cm^2)	ECSA After Sulfonamide Coupling (cm^2)
Capillary Gap	1360	472	4220	214
Rotating Cylinder (outer electrode)	3258	2938	N/A	6463
Rotating Cylinder (inner electrode)	2727	N/A	441	N/A

We believe that the large ECSAs reported relative to the geometric surface area are due to the porous nature of graphite.[19,20] Decreases in the ECSA after reaction, like in sulfonamide coupling in the capillary gap reactor, could indicate fouling of the electrodes, as we also observed by inspection of the electrodes. Increases in the ECSA after reaction, like in XEC in the capillary gap reactor, could indicate improved wetting of graphite pores after extended operation. The measurements could also be influenced by the presence of Faradaic processes taking place due to the presence of material deposited during operation.

Charge Transfer Resistance

We measured charge transfer resistances before and after reactions using electrochemical impedance spectroscopy (EIS) measurements. In these measurements, we coupled the leakless miniature Ag/AgCl reference electrode to a low impedance electrode shunt. The inclusion of the shunt, which is a platinum wire (Kurt J. Lesker) in contact with the solution close to the reference

electrode and coupled to the reference electrode through a 100 nF capacitor, was used to lower the impedance on the reference electrode channel for the high frequency measurements.[22]

The EIS curves were fitted to the equivalent circuit shown in **Figure S25**, to extract parameters to calculate the charge transfer resistance. The equivalent circuit included the solution resistance, a resistor in parallel with a constant phase element representing the working electrode interface, and a porous bounded Warburg element to represent diffusion within the porous graphite electrode.[22–25] This equivalent circuit is likely simplified compared to the complexity of the real interfaces found in the reactors, but serves as means to estimate the charge transfer resistance from the EIS measurements.

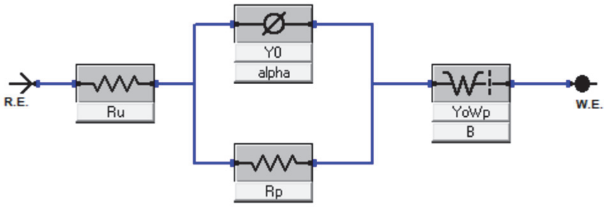

Figure S25: Equivalent circuit used to for EIS fitting.

The charge transfer resistance on the working electrodes was measured before and after each reaction, using the same electrolyte as used for the reaction. For these EIS measurements, the capillary gap reactor was assembled with only one anode and one cathode, while four of each electrodes were used when reactions were performed in between charge transfer resistance measurements. Galvanostatic EIS curves were completed in the frequency range of 0.1 Hz to 10 kHz, unless otherwise noted, and at 0.1 A, 0.2 A, and 0.3 A DC current. These EIS measurements were completed while solution was flowing at 50 mL min^{-1}, 100 mL min^{-1}, and 150 mL min^{-1}. The charge transfer resistance was calculated by extracting R_P from the equivalent circuit in **Figure S25**. The Nyquist curves before and after each reaction are given in **Figures S26-S31** and charge transfer resistances extracted for each reaction and reactor are tabulated in **Tables S6-S11**.

For the EIS measurements after mediated alcohol oxidation at 0.1 A in the capillary gap reactor, the measurements were run from 1 Hz to 10 kHz.

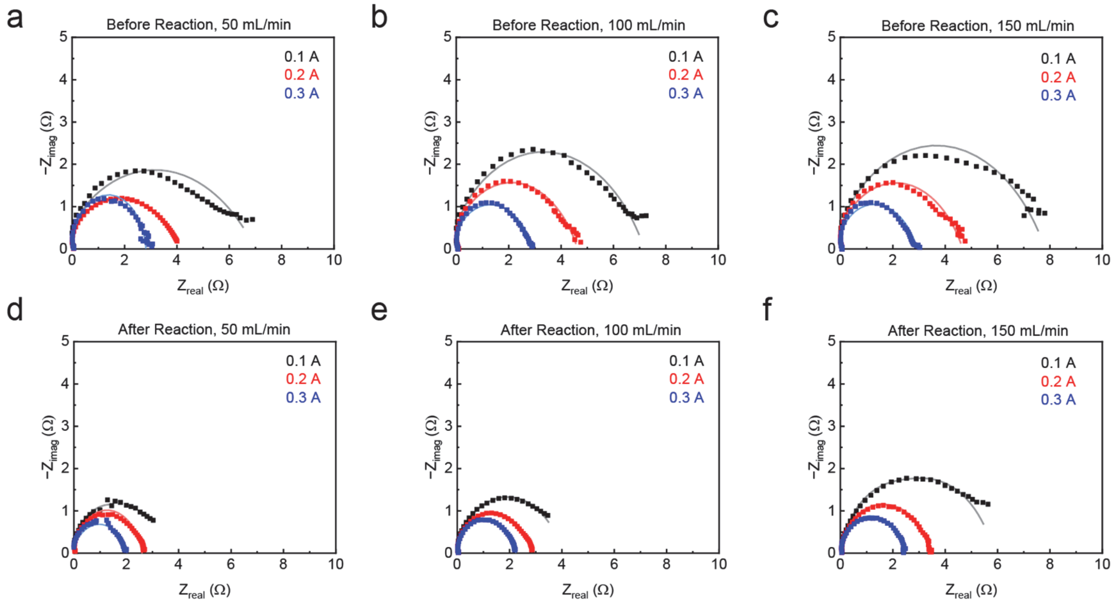

Figure S26: Nyquist curves for EIS of graphite electrodes in the capillary gap reactor before **(a-c)** and after **(d-f)** mediated alcohol oxidation. Lines shown are from fitted parameters.

Table S6: Calculated Charge Transfer Resistance for Alcohol Oxidation in Capillary Gap Reactor at Different Currents and Flow Rates

	0.1 A	0.2 A	0.3 A
	Before Reaction		
50 mL min^{-1}	7.7 Ω	4.8 Ω	2.9 Ω
100 mL min^{-1}	7.8 Ω	5.2 Ω	3.4 Ω
150 mL min^{-1}	8.4 Ω	5.2 Ω	3.4 Ω
	After Reaction		
50 mL min^{-1}	5.4 Ω	3.0 Ω	2.1 Ω
100 mL min^{-1}	4.3 Ω	3.1 Ω	2.5 Ω
150 mL min^{-1}	8.4 Ω	4.7 Ω	3.3 Ω

For the EIS measurements before XEC at 0.1 A in the capillary gap reactor, the measurements were run from 1 Hz to 10 kHz.

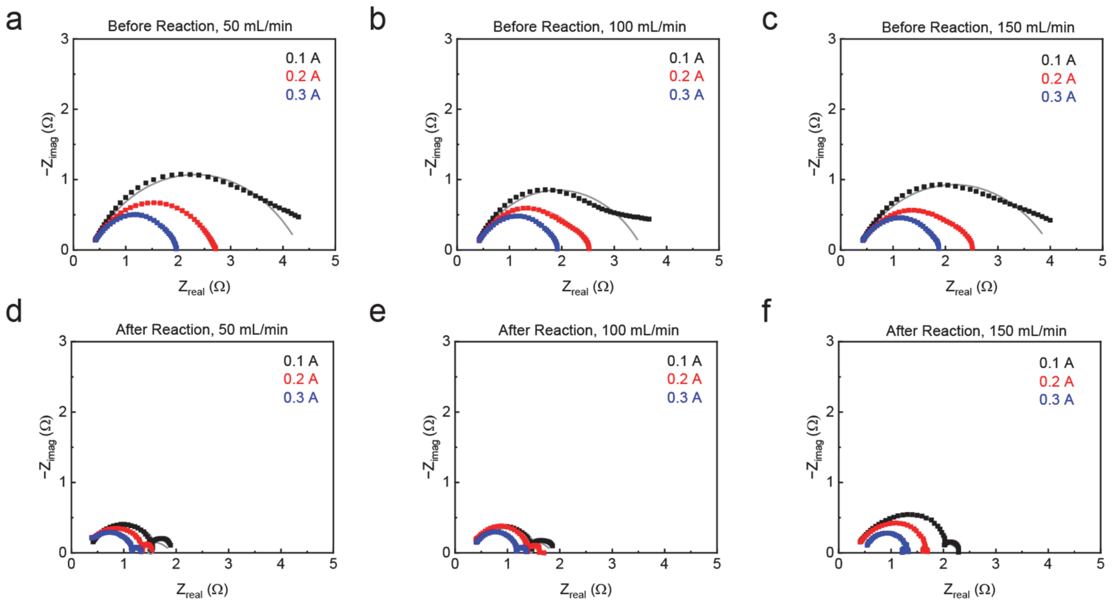

Figure S27: Nyquist curves for EIS of graphite electrodes in the capillary gap reactor before **(a-c)** and after **(d-f)** XEC. Lines shown are from fitted parameters.

Table S7: Calculated Charge Transfer Resistances for XEC in Capillary Gap Reactor at Different Currents and Flow Rates

	0.1 A	0.2 A	0.3 A
	Before Reaction		
50 mL min^{-1}	4.0 Ω	2.4 Ω	1.7 Ω
100 mL min^{-1}	3.7 Ω	2.2 Ω	1.6 Ω
150 mL min^{-1}	3.8 Ω	2.3 Ω	1.6 Ω
	After Reaction		
50 mL min^{-1}	2.9 Ω	2.2 Ω	1.7 Ω
100 mL min^{-1}	2.7 Ω	2.3 Ω	1.6 Ω
150 mL min^{-1}	2.9 Ω	1.8 Ω	1.4 Ω

For the EIS measurements after sulfonamide coupling at 0.1 A in the capillary gap reactor, the measurements were run from 1 Hz to 10 kHz.

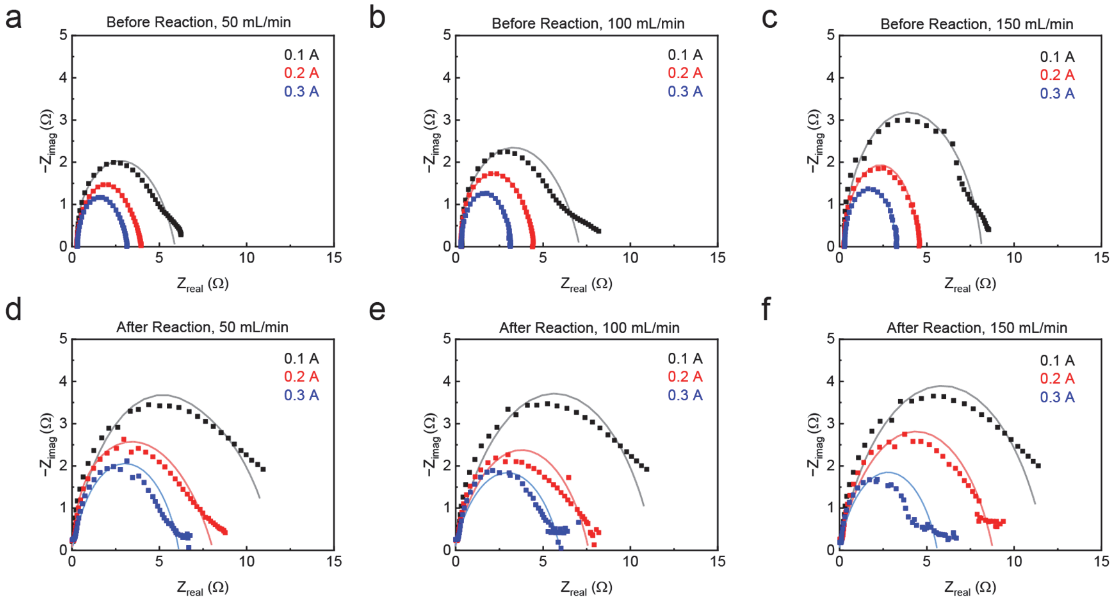

Figure S28: Nyquist curves for EIS of graphite electrodes in the capillary gap reactor before **(a-c)** and after **(d-f)** sulfonamide coupling. Lines shown are from fitted parameters.

Table S8: Calculated Charge Transfer Resistances for Sulfonamide Coupling in Capillary Gap Reactor at Different Currents and Flow Rates

	−0.1 A	−0.2 A	−0.3 A
	Before Reaction		
50 mL min^{-1}	6.5 Ω	5.6 Ω	3.8 Ω
100 mL min^{-1}	7.8 Ω	4.5 Ω	3.0 Ω
150 mL min^{-1}	10.6 Ω	4.8 Ω	3.3 Ω
	After Reaction		
50 mL min^{-1}	14.3 Ω	10.2 Ω	5.7 Ω
100 mL min^{-1}	11.4 Ω	7.7 Ω	6.1 Ω
150 mL min^{-1}	11.8 Ω	8.8 Ω	5.6 Ω

For some measurements in the rotating cylinder reactor, instability was observed for high frequency data points. We attribute those to the electrical connections to the reactor and not to electrode processes themselves. These datapoints were therefore removed from the fits.

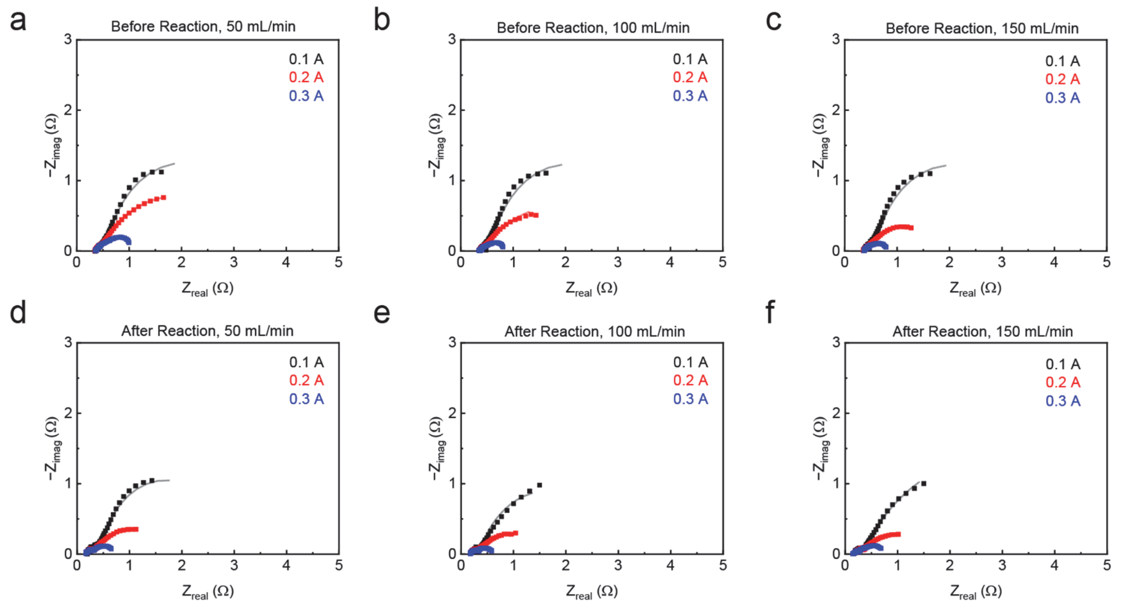

Figure S29: Nyquist curves for EIS of graphite electrodes in the rotating cylinder reactor before **(a-c)** and after **(d-f)** mediated alcohol oxidation. Lines shown are from fitted parameters.

Table S9: Calculated Charge Transfer Resistances for Alcohol Oxidation in Rotating Cylinder Reactor at Different Currents and Flow Rates

	0.1 A	0.2 A	0.3 A
		Before Reaction	
50 mL min^{-1}	2.9 Ω	2.8 Ω	0.75 Ω
100 mL min^{-1}	2.8 Ω	1.3 Ω	0.63 Ω
150 mL min^{-1}	2.8 Ω	1.7 Ω	0.55 Ω
		After Reaction	
50 mL min^{-1}	2.5 Ω	1.6 Ω	0.66 Ω
100 mL min^{-1}	2.6 Ω	1.3 Ω	0.38 Ω
150 mL min^{-1}	4.8 Ω	1.2 Ω	0.72 Ω

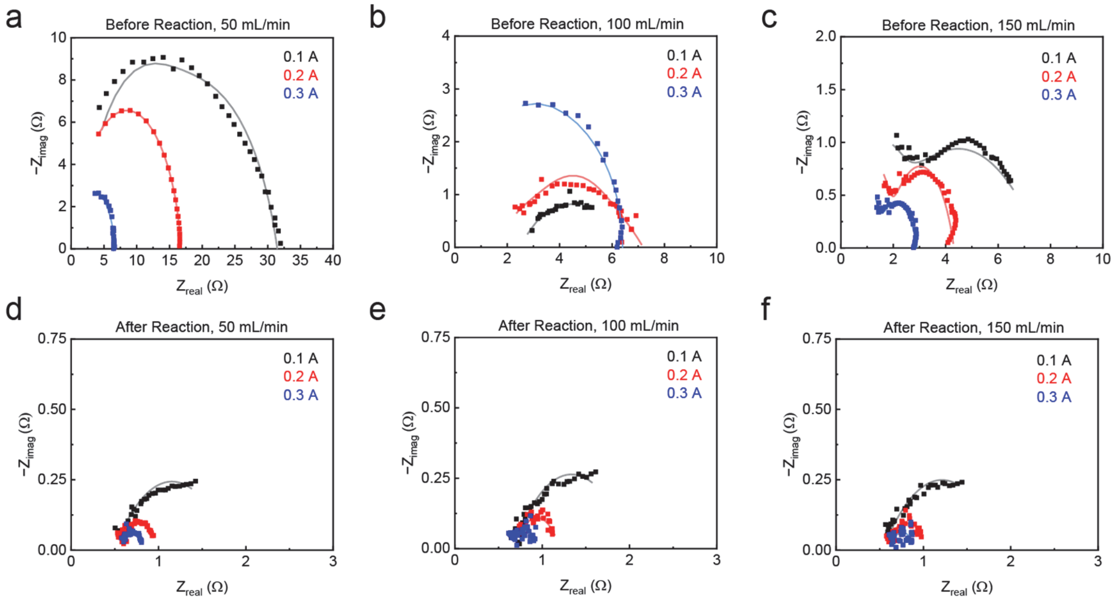

Figure S30: Nyquist curves for EIS of graphite electrodes in the rotating cylinder reactor before **(a-c)** and after **(d-f)** XEC. Lines shown are from fitted parameters.

Table S10: Calculated Charge Transfer Resistances for XEC in Rotating Cylinder Reactor at Different Currents and Flow Rates

	0.1 A	0.2 A	0.3 A
	Before Reaction		
50 mL min^{-1}	19.6 Ω	17.2 Ω	6.8 Ω
100 mL min^{-1}	4.1 Ω	4.6 Ω	4.3 Ω
150 mL min^{-1}	6.2 Ω	2.5 Ω	1.6 Ω
	After Reaction		
50 mL min^{-1}	1.3 Ω	0.67 Ω	0.28 Ω
100 mL min^{-1}	1.3 Ω	0.51 Ω	0.41 Ω
150 mL min^{-1}	1.3 Ω	0.52 Ω	0.32 Ω

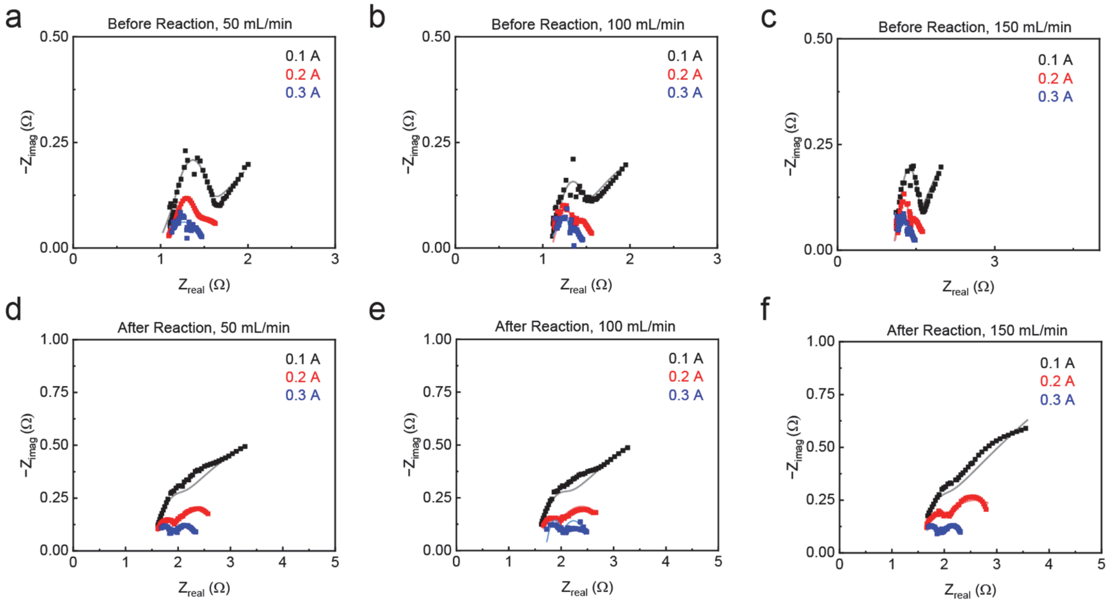

Figure S31: Nyquist curves for EIS of graphite electrodes in the rotating cylinder reactor before **(a-c)** and after **(d-f)** sulfonamide coupling. Lines shown are from fitted parameters.

Table S11: Calculated Charge Transfer Resistances for Sulfonamide Coupling in Rotating Cylinder Reactor at Different Currents and Flow Rates

	0.1 A	0.2 A	0.3 A
	Before Reaction		
50 mL min^{-1}	5.4 Ω	0.65 Ω	0.50 Ω
100 mL min^{-1}	2.0 Ω	0.38 Ω	0.42 Ω
150 mL min^{-1}	2.2 Ω	0.54 Ω	0.44 Ω
	After Reaction		
50 mL min^{-1}	5.7 Ω	1.4 Ω	0.69 Ω
100 mL min^{-1}	8.3 Ω	1.4 Ω	0.57 Ω
150 mL min^{-1}	16.1 Ω	1.9 Ω	0.83 Ω

Overall, the reported charge transfer resistances do not change significantly with flow rate. The charge transfer resistance decreases at higher applied currents, and in most cases, they do not change significantly after a reaction. The charge transfer resistances increase after sulfonamide coupling in the capillary gap reactor and at 0.1 A in the rotating cylinder reactor, which could be indicative of fouling on the electrode surface or by consumption of all electroactive species during the course of the sulfonamide coupling reaction, as this is the only reaction where a mediator is not regenerated. We believe that that the minimal changes in charge transfer resistance for most reactions likely indicate that electrochemical activity under DC currents is primarily confined to the surface of the electrodes as defined by their geometry and does not involve the depth of the porous graphite network.

Calculating Production Rates

For single-pass reactions, total production of material in mmol at each time point was calculated by the following equation.

$$\sum C * Q * t$$

Where:

C = measured concentration of product in the sample in mmol/L

Q = flow rate in L/min

t = time since previous sample point in minutes

Literature Reports at Single-Pass

We sought to identify the best single-pass performance of each reaction class found in the literature to compare to the single-pass performance achieved herein. The details of these literature reports, showcased in **Figure 7** of the main text, are given below.

Table S12. Literature Reports at Single-Pass

Reaction	Reactor Type	Electrode Surface Area (cm^2)	Product Yield (%)	Production Rate (mmol/h)	Scale (mmol)
Mediated Alcohol Oxidation[26]	Microfluidic flow cell	10	92	0.56	0.47
XEC[27]	Parallel plate flow cell	9.6	72	0.15	0.22
Sulfonamide Coupling[17]	Microflow cell	26	69	0.62	1.4

References

1. Malkowsky, I. M. *et al.* Novel Template-Directed Anodic Phenol-Coupling Reaction. *Chemistry - A European Journal* **12**, 7482–7488 (2006).
2. Jüttner, K. Technical Scale of Electrochemistry. in *Encyclopedia of Electrochemistry* (Wiley, 2007). doi:10.1002/9783527610426.bard050001.
3. Cai, Z., Liu, B., Zou, X. & Cheng, H.-M. Chemical Vapor Deposition Growth and Applications of Two-Dimensional Materials and Their Heterostructures. *Chem Rev* **118**, 6091–6133 (2018).
4. Physical Vapor Deposition. in *The Materials Science of Semiconductors* 505–572 (Springer US, Boston, MA, 2008). doi:10.1007/978-0-387-68650-9_11.
5. Gurrappa, I. & Binder, L. Electrodeposition of nanostructured coatings and their characterization—A review. *Sci Technol Adv Mater* **9**, 043001 (2008).
6. Barker, B. D. Electroless deposition of metals. *Surface Technology* **12**, 77–88 (1981).
7. Bird, R. B., Stewart, W. E. & Lightfoot, E. N. *Transport Phenomena*. (John Wiley & Sons, Inc., New York, 2002).
8. Detry, J. G., Deroanne, C., Sindic, M. & Jensen, B. B. B. Laminar flow in radial flow cell with small aspect ratios: Numerical and experimental study. *Chem Eng Sci* **64**, 31–42 (2009).
9. Moller, P. S. Radial Flow without Swirl between Parallel Discs. *Aeronautical Quarterly* **14**, 163–186 (1963).
10. Nemri, M., Charton, S. & Climent, E. Mixing and axial dispersion in Taylor–Couette flows: The effect of the flow regime. *Chem Eng Sci* **139**, 109–124 (2016).
11. Schrimpf, M. *et al.* Taylor-Couette reactor: Principles, design, and applications. *AIChE Journal* **67**, (2021).
12. Frenkel, D. & Smit, B. *Understanding Molecular Simulation: From Algorithms to Applications*. vol. 2nd Edition (2002).
13. Hills, E. E., Abraham, M. H., Hersey, A. & Bevan, C. D. Diffusion coefficients in ethanol and in water at 298K: Linear free energy relationships. *Fluid Phase Equilib* **303**, 45–55 (2011).
14. Hayashi, K., Griffin, J., Harper, K. C., Kawamata, Y. & Baran, P. S. Chemoselective (Hetero)Arene Electroreduction Enabled by Rapid Alternating Polarity. *J Am Chem Soc* **144**, 5762–5768 (2022).
15. Rafiee, M., Konz, Z. M., Graaf, M. D., Koolman, H. F. & Stahl, S. S. Electrochemical Oxidation of Alcohols and Aldehydes to Carboxylic Acids Catalyzed by 4-Acetamido-TEMPO: An Alternative to "Anelli" and "Pinnick" Oxidations. *ACS Catal* **8**, 6738–6744 (2018).
16. Perkins, R. J., Pedro, D. J. & Hansen, E. C. Electrochemical Nickel Catalysis for Sp2-Sp3 Cross-Electrophile Coupling Reactions of Unactivated Alkyl Halides. *Org Lett* **19**, 3755–3758 (2017).
17. Laudadio, G. *et al.* Sulfonamide Synthesis through Electrochemical Oxidative Coupling of Amines and Thiols. *J Am Chem Soc* **141**, 5664–5668 (2019).

18. Connor, P., Schuch, J., Kaiser, B. & Jaegermann, W. The Determination of Electrochemical Active Surface Area and Specific Capacity Revisited for the System MnO $_x$ as an Oxygen Evolution Catalyst. *Zeitschrift für Physikalische Chemie* **234**, 979–994 (2020).
19. Smith, R. E. G., Davies, T. J., Baynes, N. de B. & Nichols, R. J. The electrochemical characterisation of graphite felts. *Journal of Electroanalytical Chemistry* **747**, 29–38 (2015).
20. Calas-Blanchard, C., Comtat, M., Marty, J.-L. & Mauran, S. Textural characterisation of graphite matrices using electrochemical methods. *Carbon N Y* **41**, 123–130 (2003).
21. Yoon, Y., Yan, B. & Surendranath, Y. Suppressing Ion Transfer Enables Versatile Measurements of Electrochemical Surface Area for Intrinsic Activity Comparisons. *J Am Chem Soc* **140**, 2397–2400 (2018).
22. Mansfeld, F., Lin, S., Chen, Y. C. & Shih, H. Minimization of High-Frequency Phase Shifts in Impedance Measurements. *J Electrochem Soc* **135**, 906–907 (1988).
23. Macdonald, J. R. Impedance spectroscopy. *Ann Biomed Eng* **20**, 289–305 (1992).
24. Cruz-Manzo, S. & Greenwood, P. An impedance model based on a transmission line circuit and a frequency dispersion Warburg component for the study of EIS in Li-ion batteries. *Journal of Electroanalytical Chemistry* **871**, 114305 (2020).
25. de Levie, R. & Pospíšil, L. On the coupling of interfacial and diffusional impedances, and on the equivalent circuit of an electrochemical cell. *J Electroanal Chem Interfacial Electrochem* **22**, 277–290 (1969).
26. Hill-Cousins, J. T. *et al.* TEMPO-Mediated Electrooxidation of Primary and Secondary Alcohols in a Microfluidic Electrolytic Cell. *ChemSusChem* **5**, 326–331 (2012).
27. Franke, M. C. *et al.* Zinc-free, Scalable Reductive Cross-Electrophile Coupling Driven by Electrochemistry in an Undivided Cell. *ACS Catal* **12**, 12617–12626 (2022).

Representative NMR Spectra

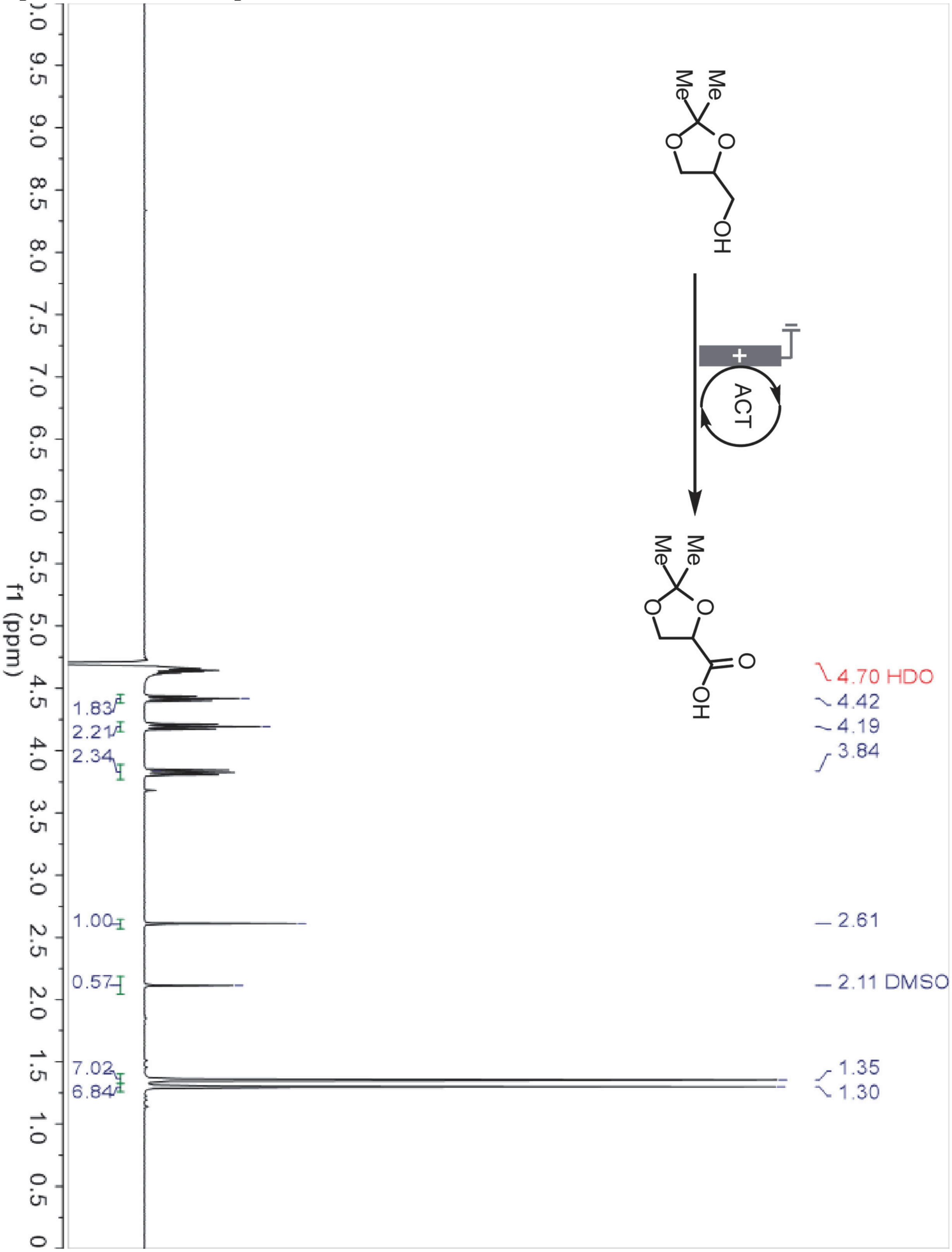

Figure S32: ^{1}H NMR spectrum showing reaction solution for a representative alcohol oxidation reaction taken in D_2O solvent.

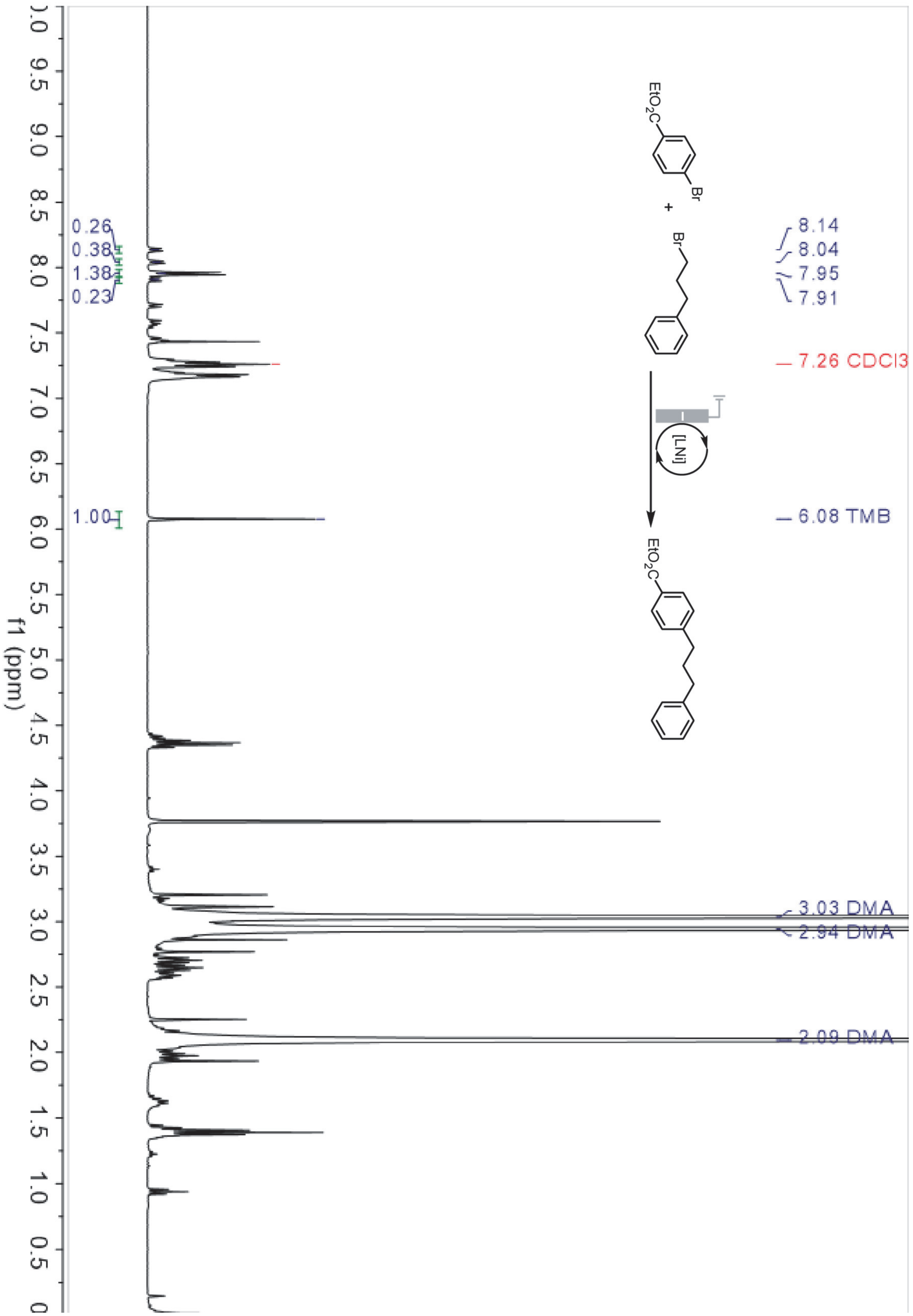

Figure S33: ^{1}H NMR spectrum showing reaction solution for a representative Ni-catalyzed XEC reaction taken in $CDCl_3$ solvent.

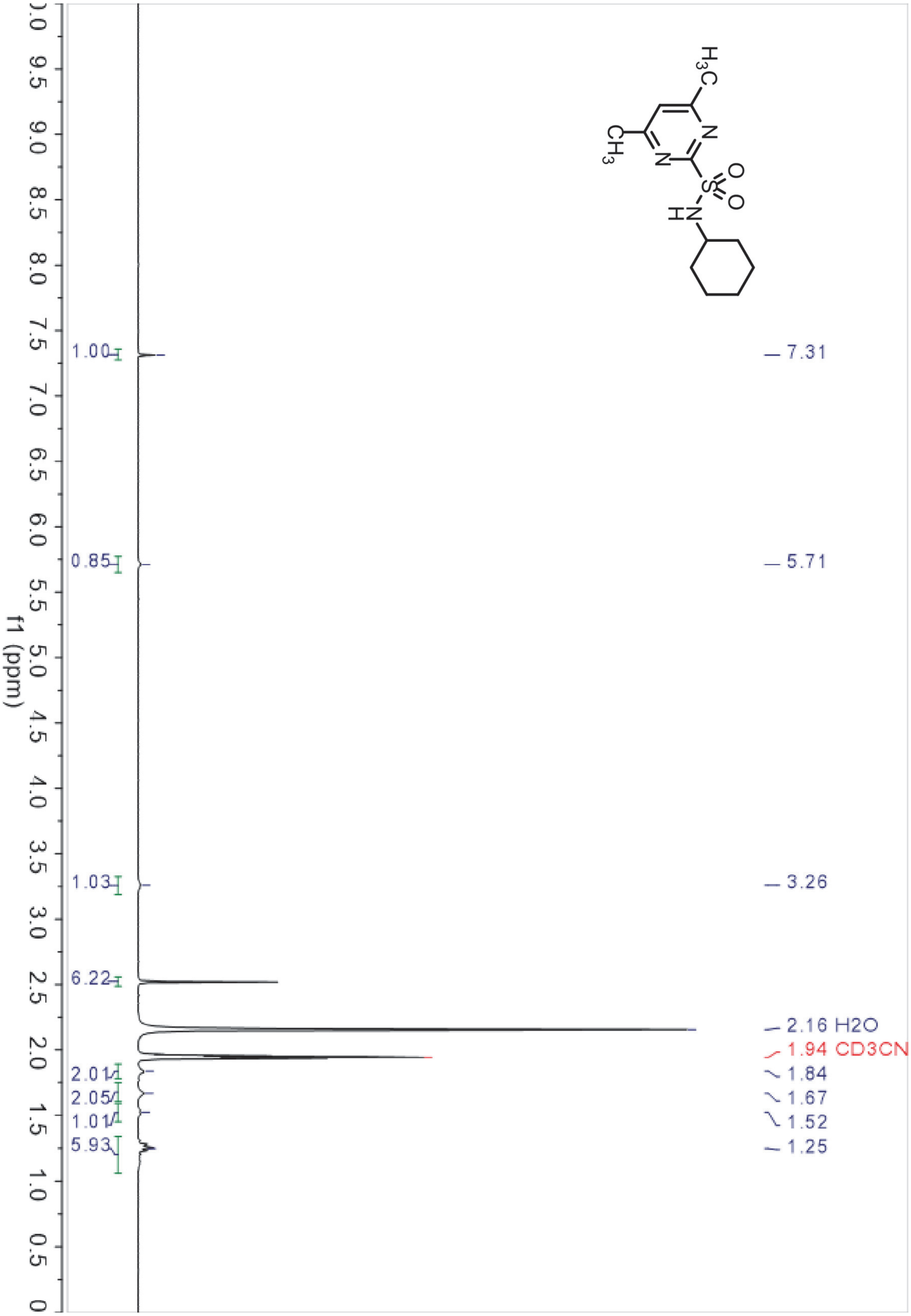

Figure S34: ^{1}H NMR spectrum of the primary sulfonamide product from the sulfonamide coupling reaction taken in CD_3CN solvent.

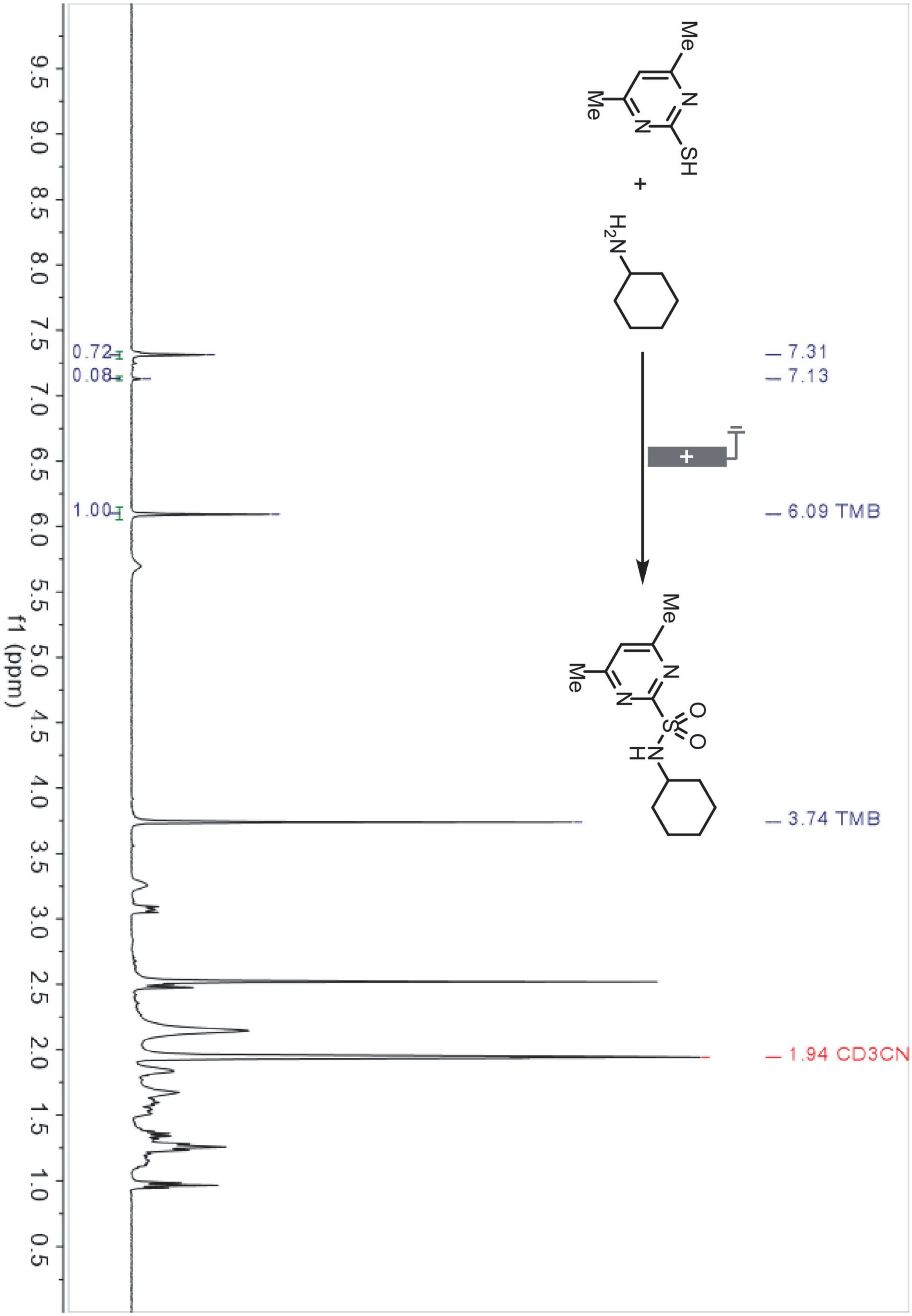

Figure S35: ^{1}H NMR spectrum showing reaction solution for a representative sulfonamide coupling reaction taken in CD_3CN solvent.
